# Cyclosporine A Accelerates Neurorecovery Transcriptional Trajectory in a Swine Model of Diffuse Traumatic Brain Injury

**DOI:** 10.3390/ijms26083531

**Published:** 2025-04-09

**Authors:** Oluwagbemisola Aderibigbe, Levi B. Wood, Susan S. Margulies

**Affiliations:** 1 Wallace H. Coulter Department of Biomedical Engineering, Georgia Institute of Technology and Emory University, Atlanta, GA 30332, USA; oaderibigbe7@gatech.edu; 2 George W. Woodruff School of Mechanical Engineering and Parker H. Petit Institute for Bioengineering and Bioscience, Georgia Institute of Technology, Atlanta, GA 30332, USA

**Keywords:** TBI, RNA-seq, gene expression, weighted gene co-expression network analysis, pediatric, treatment, pig

## Abstract

Mild traumatic brain injury (mTBI) is a leading cause of morbidity in children with both short- and long-term neurological, cognitive, cerebrovascular, and emotional deficits. These deficits have been attributed to ongoing pathophysiological cascades that occur acutely and persist post-injury. Given our limited understanding of the transcriptional changes associated with these pathophysiological cascades, we studied formalin-fixed paraffin-embedded (FFPE) tissues from the frontal cortex (FC) and the hippocampus + amygdala (H&A) regions of swine (N = 40) after a sagittal rapid non-impact head rotation (RNR). We then sequenced RNA to define transcriptional changes at 1 day and 1 week after injury and investigated the protective influence of cyclosporine A (CsA) treatment. Differentially expressed genes (DEGs) were classified into five temporal patterns (Early, Transient, Persistent, Intensified, Delayed, or Late). DEGs were more abundant at 1 week than 1 day. Shared significant gene ontology annotations in both regions included terms associated with neuronal distress at 1 day and neurorecovery at 1 week. CsA (20 mg/kg/day) infused for 1 day (beginning at 6 h after injury) accelerated 466 DEGs in the FC and 2794 DEGs in the H&A, such that the CsA-treated transcriptional profile was associated with neurorecovery. Overall, our data reveal the effects of anatomic region and elapsed time on gene expression post-mTBI and motivate future studies of CsA treatment.

## 1. Introduction

Traumatic brain injury (TBI) is a leading cause of death and disability in adults and young children [1,2]. In the United States, millions of people suffer TBI yearly, with infant/toddler (0–4 years), adolescent (15–19 years), and elderly (>65 years) groups having the highest incidences of TBI [3,4]. Approximately 80% of all TBI cases are classified as mild. Among mild cases, 15–30% of mild TBI (mTBI) patients have complicated recoveries that can last months to years post-injury [5,6]. Extended duration of recovery is particularly concerning for the pediatric population, whose brains are under development and are thus more likely to develop prolonged cognitive, social, and emotional impairments [7,8,9]. Given the vulnerability of this population to mTBI, the goal of the current study was to utilize transcriptional profiling to holistically define tissue-level changes in a pediatric swine model of rotational mTBI.

Defining when and where genes and pathways are modulated following mTBI is essential to understanding mTBI progression and the design of targeted novel therapeutics. While numerous prior TBI studies have identified neuronal and immune transcriptional profiles changing after injury [10,11,12,13,14,15,16,17,18], fewer have focused on pediatric TBI [19,20,21,22] or identified differentially expressed genes (DEGs) based on sex, time elapsed, and injury severity in various regions of the brain.

We previously examined cyclosporine A (CsA) as an mTBI post-injury therapy and found that it improves outcomes [23,24,25]. CsA is an FDA-approved, off-patent, inexpensive drug that has been utilized in several open clinical trials in pediatric disorders [23]. There are two well-known mechanisms of action for CsA. Firstly, it inhibits the cyclophilin D (CypD)-dependent and calcium-mediated activation of mPTP to attenuate apoptosis [26,27]. Secondly, it decreases the release of pro-inflammatory mediators by (i) inhibiting the NF-κB pathway to prevent the transcription of pro-inflammatory cytokines [28], (ii) by binding to cyclophilin (CyP) to inhibit calcineurin-mediated de-phosphorylation of nuclear factor of activated T cells (NFAT) and the transcription of cytokines, like IL-2 and IL-4 [28,29], or (iii) by inhibiting the activation of MKK6 and MKK7 to block p38 and JNK activation [30,31]. Although it has well-described safety and dosing profiles, few TBI studies have focused on its potential for improving TBI outcomes [32,33,34]. Previously, we demonstrated the pleiotropic effects of CsA in improving TBI outcomes in a dose-dependent manner in 4-week-old piglets who received a sagittal rapid non-impact head rotation (RNR) or controlled cortical impact (CCI), with decreasing brain lesion volume, increasing mitochondrial function, and decreasing apoptosis and pro-inflammatory markers [23,24,25]. When considering efficacy for both CCI and RNR, we showed that a 20 mg/kg/day CsA dose administered, beginning with a bolus at 6 h post-injury and continuous infusion for 1 day, was the optimal dose for (i) increasing the mitochondrial respiratory control ratio (RCR) and (ii) decreasing injured brain volume (pathology) compared with other doses and other delay conditions evaluated [24]. There are no studies reporting CsA’s effects on gene expression following diffuse mTBI in a porcine preclinical model of diffuse mTBI [35,36]. Due to the abundance of prior literature investigating CsA in both injury and Sham experimental groups, including demonstrating the upregulation of regenerative genes in healthy brains, we focused the current study on the potential therapeutic benefits of CsA in the setting of injury.

In this present study, we hypothesized that the transcriptional profiles in the brain would change as a function of time post-mTBI, and that CsA infusion after mTBI (20 mg/kg/day) would promote changes in genes associated with neurorecovery. Neurorecovery DEGs and processes are defined as sets of genes or processes that play a crucial role in the repair and recovery of the brain following injury [37,38]. We tested this by using a sagittal rapid non-impact head rotation (RNR) swine mTBI model and RNA sequencing analysis of previously collected formalin-fixed, paraffin-embedded (FFPE) tissues from both the frontal cortex (FC) and the hippocampus + amygdala (H&A). These brain regions were evaluated due to their roles in driving crucial functions, like behavior, cognition, memory, and socio-emotional functioning, which are usually affected by TBI [39,40,41,42]. Moreover, the frontal cortex is known to be particularly vulnerable to TBI [43]. Collectively, our data reveal pronounced changes in gene expression with elapsed time after mTBI and highlight the role of CsA in accelerating changes in gene expression associated with neurorecovery.

## 2. Results

### 2.1. Brain Transcriptional Alterations Follow Six Temporal Patterns Between 1 Day and 1 Week Post-Injury

**Figure 1:**
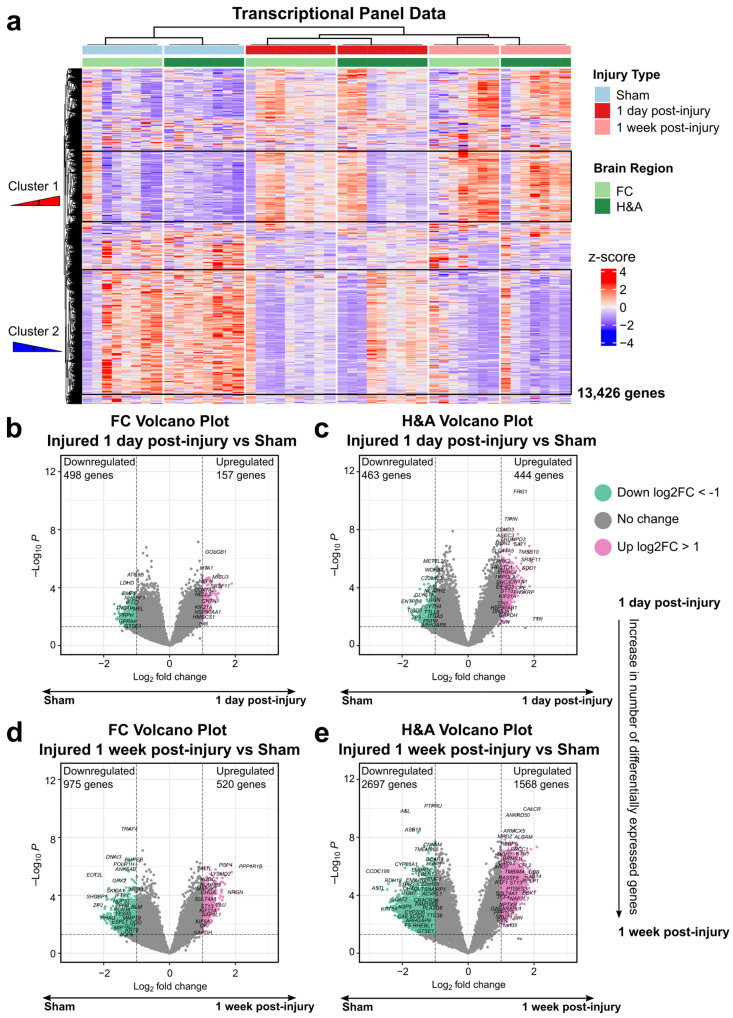
Increased alteration of genes by 1 week post-injury in both regions. (**a**) Hierarchal clustering of all genes across all injury groups in the FC (light green) and H&A (dark green). Each row represents a gene, and each column represents an animal subject (rows were z-scored). (**b**) Differentially expressed genes (DEGs) between the Sham and injured 1 day post-injury groups in the FC and (**c**) H&A, consecutively. DEGs have unadjusted *p*-values ≤ 0.05 (above dashed horizontal line) and corresponding log2 fold change |log2FC| ≥ 1. (**d**) DEGs between the Sham and injured 1 week post-injury groups in the FC and (**e**) H&A, consecutively. DEGs have unadjusted *p*-values ≤ 0.05 (above dashed horizontal line) and corresponding log2 fold change |log2FC| ≥ 1. mTBI, mild traumatic brain injury. DEGs, differentially expressed genes. Log2FC, log2 fold change. FC, frontal cortex. H&A, hippocampus + amygdala.

We began by evaluating our hypothesis regarding the effects of injury and time elapsed after mTBI on gene expression profiles in each brain region. To do so, we performed bulk RNA sequencing (RNA-seq) on formalin-fixed paraffin-embedded (FFPE) brain tissues from frontal cortex (FC) and the hippocampus + amygdala (H&A) brain regions in three experimental groups spanning 13,426 genes: Sham, 1 day post-injury, and 1 week post-injury. Hierarchal clustering identified two clusters of genes displaying variation in expression between all three groups (Figure 1a). Cluster 1 included genes with a progressive increase in expression from Sham to 1 day post-injury to 1 week post-injury, while Cluster 2 included genes with progressive decrease from Sham to 1 day post-injury to 1 week post-injury. The H&A displayed more subject-to-subject heterogeneity in gene expression at 1 day post-injury compared to the FC, in addition to more uniform responses at 1 week post-injury. Consistent with these general trends, there was also an increased number of differentially expressed genes (DEGs) (*p* ≤ 0.05, log2FC ≥ 1 or ≤ −1) from 1 day post-injury (Figure 1b,c) to 1 week post-injury (Figure 1d,e) in both regions, more dramatically within the H&A. This was also observed in DEGs selected using FDR ≤ 0.05, |log2FC| ≥ 1 (Supplementary Figure S1). Thus, the injury resulted in a progressive increase in numbers of DEGs from 1 day to 1 week post-injury, especially in the H&A.

**Figure 2:**
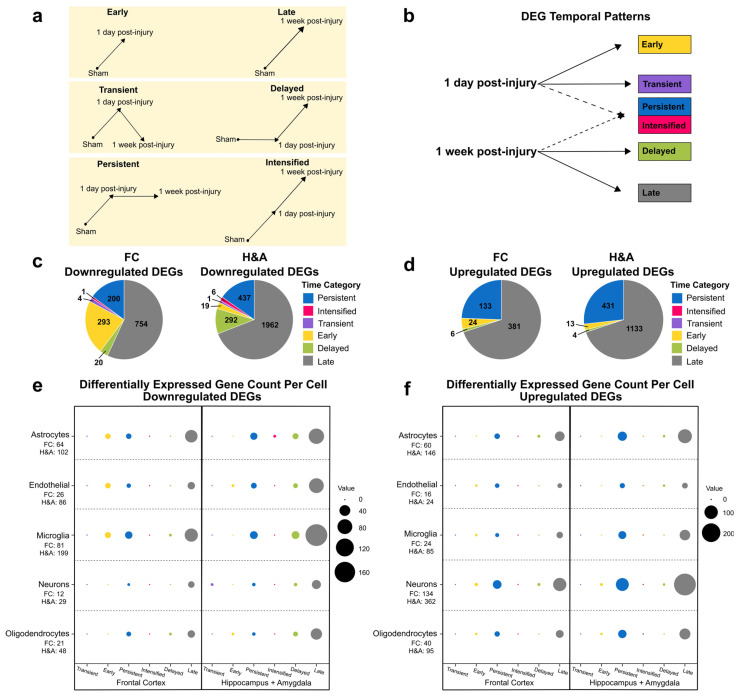
Temporal patterns for differentially expressed genes. (**a**) DEG temporal pattern definitions and descriptions. Early: DEG only at 1 day compared to Sham. Transient: DEG only at 1 day compared to both Sham and 1 week post-injury. Persistent: DEG at 1 day and remained significantly altered by 1 week post-injury, both compared to Sham. Intensified: DEG at 1 day, and significantly altered further at 1 week post-injury relative to the 1-day level. Delayed: DEG only at 1 week post-injury, when compared to both Sham and 1 day post-injury. Late: DEG only at 1 week post-injury compared to Sham. (**b**) Connection between post-injury timepoints and temporal patterns. (**c**) Populations of Transient, Early, Persistent, Intensified, Delayed, and Late downregulated DEGs. (**d**) Populations of Persistent, Intensified, Transient, Early, Delayed, and Late upregulated DEGs. (**e**) Number of cell type-specific genes within each downregulated temporal pattern. The total number of DEGs for each specific cell type in FC and H&A are listed on the vertical axis. (**f**) Number of cell-specific genes within each upregulated temporal pattern. The total number of DEGs for each specific cell type in FC and H&A are listed on the vertical axis. The size of the circles represents the number of cell type-specific genes from each cell (vertical axis) in each temporal pattern (horizontal axis). DEGs, differentially expressed genes. FC, frontal cortex. H&A, hippocampus + amygdala.

To distinguish post-injury timing patterns, we next sorted all significant DEGs (*p* ≤ 0.05, |log2FC| ≥ 1) into six temporal patterns (Figure 2a,b). Summing up all DEGs within these temporal patterns will produce the total number of DEGs in Figure 1.

1. **Early:** DEG only at 1 day compared to Sham.
2. **Transient:** DEG only at 1 day compared to both Sham and 1 week post-injury.
3. **Persistent:** DEG at 1 day and remained significantly altered by 1 week post-injury, both compared to Sham.
4. **Intensified:** DEG at 1 day and significantly altered further at 1 week post-injury relative to the 1-day level.
5. **Delayed:** DEG only at 1 week post-injury, when compared to both Sham and 1 day post-injury.
6. **Late:** DEG only at 1 week post-injury compared to Sham.

The majority of the DEGs were Late (Figure 2c,d). For downregulated DEGs in the FC, Early, Persistent, and Late downregulated DEGs were most abundant, while for downregulated DEGs in the H&A, Persistent, Delayed, and Late downregulated DEGs were most abundant (Figure 2c). The Intensified or Transient DEGs in either region were all downregulated. Further, the H&A had more Delayed downregulated DEGs than the FC, while the FC had more Early downregulated DEGs compared to the H&A. Thus, there were more Early gene changes in the FC and more Delayed gene changes in the H&A. In both regions, most of the upregulated DEGs were Persistent or Late (Figure 2d).

After identifying the numbers of DEGs within our six temporal pattern groups, we categorized which cell types were associated with these temporal patterns. We focused on astrocytes, endothelial cells, microglia, neurons, and oligodendrocyte cells, because they are the most common cells in the brain. Genes with high specificity to each of these cells (cell type-specific genes) were obtained by calculating a cell-type enrichment τ score as outlined in Galea et al., 2022 [44]. Genes with τ ≥ 0.8 for a specific cell were identified as being specific to that cell and utilized in this analysis.

Among downregulated DEGs, we discovered that in the FC, the Early, Persistent, and Late DEGs were predominantly astrocyte- and microglia-specific (Figure 2e, Supplementary Table S2). In the H&A, the Persistent, Delayed, and Late DEGs were predominantly astrocyte-, endothelial-, and microglia-specific (Figure 2e, Supplementary Table S2). Among upregulated DEGs, we noted more consistency between regions; specifically, the Persistent and Late DEGs were mainly astrocyte- and neuron-specific (Figure 2f, Supplementary Table S2). By focusing on the two largest DEG timing patterns (Persistent and Late), we observed a downregulation of mainly microglia-, endothelia-, and astrocyte-specific DEGs and upregulation of mainly neuron- and astrocyte-specific DEGs. Interestingly, of all DEGs at 1 week post-injury, microglia-specific DEGs were mostly downregulated, while neuron-specific DEGs were mostly upregulated.

### 2.2. Genes Associated with Neurodegeneration, Immune Signaling, Synaptic Signaling, and Development Are Altered at 1 Day Post-Injury

Once we established the temporal patterns and cell affinities of all DEGs, we then characterized DEG functions dominant at 1 day post-injury, both by using GO overrepresentation analysis and by examining the functions of specific DEGs. We present the four temporal patterns in the following order: (i) Transient, (ii) Early, (iii) Intensified, and (iv) Persistent. Within each temporal pattern, we will then focus on downregulated followed by upregulated DEGs.

**Figure 3:**
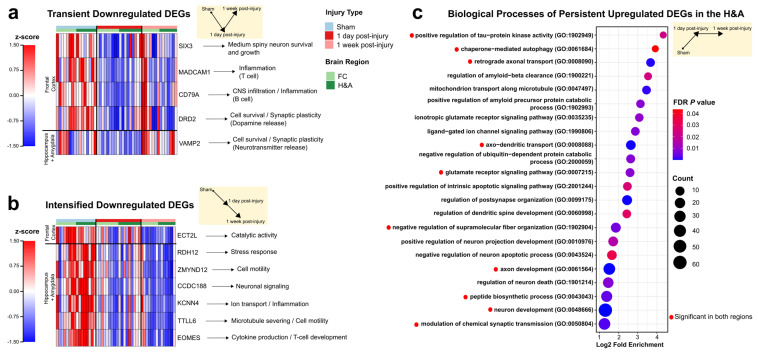
Neuronal and immune genes are altered at 1 day post-injury. (**a**) Transient downregulated DEGs. Each row represents a DEG, and each column represents an animal subject (rows are z-scored). (**b**) Intensified downregulated DEGs. Each row represents a DEG, and each column represents an animal subject (rows were z-scored). (**c**) Top GO terms for the Persistent upregulated DEGs in the H&A (FDR ≤ 0.05). Red circles indicate those significant in the FC and H&A. Red to blue indicates high to low FDR *p*-values. Count is the number of DEGs making up each GO term. DEGs, differentially expressed genes. FDR, false discovery rate. FC, frontal cortex. H&A, hippocampus + amygdala.

For Transient DEGs, PANTHER revealed no significant overrepresented GO terms. However, the four Transient downregulated DEGs in the FC and the 1 Transient downregulated DEG in the H&A are involved in immune and/or neuronal functions. In particular, *SIX3*, *MADCAM1*, *CD79A*, and *DRD2* were Transient downregulated DEGs in the FC and are known for their roles in neuron survival, production, and growth (*SIX3*) [45,46,47], CNS inflammation and endothelial cell adhesion (*MADCAM1*) [48,49], CNS infiltration and B cell function (*CD79A*) [50], and synaptic signaling (*DRD2*) [51] (Figure 3a, Supplementary Table S3, Tab 1). As discussed later in the results, *SIX3* was one of the top 5 downregulated DEGs in the FC at 1 day post-injury. *VAMP2*, which was the only Transient downregulated DEG in the H&A, plays a major role in synaptic function by mediating neurotransmitter release [52,53,54] (Figure 3a, Supplementary Table S3, Tab 2). There were no Transient upregulated DEGs in either region; all were downregulated DEGs. In sum, Transient downregulated DEGs were associated with immune signaling, survival, and synaptic signaling.

For Early DEGs, there were no significant overrepresented GO terms. However, among the 293 Early downregulated DEGs in the FC and 19 Early downregulated DEGs in the H&A, only *RPL3L,* known for its role in decreasing myoblast fusion [55], was shared among both regions. Of the 293 Early downregulated DEGs in the FC, the top 10 downregulated DEGs with the highest log2FC are involved in positively regulating cell adhesion, actin cytoskeletal organization, cilium assembly, ion homeostasis, cell survival, synaptic signaling, and nerve cell formation, and inhibiting glial hyperactivation (Supplementary Table S3, Tab 3). Similarly, in the H&A, the top 10 Early downregulated DEGs with the highest log2FC are involved in positively regulating neuronal survival, neuron differentiation, dopamine neurons, synaptic signaling, anti-inflammation, neuroprotection, and protein localization (Supplementary Table S3, Tab 4). These findings suggest similar functions of Early and Transient downregulated DEGs consistent with decreased cell survival, synaptic signaling, and immune suppressive responses.

For Intensified DEGs, there were no significant overrepresented GO terms. Of the one Intensified downregulated DEG in the FC (Figure 3b, Supplementary Table S3, Tab 5), and the six Intensified downregulated DEGs in the H&A (Figure 3b, Supplementary Table S3, Tab 6), three specific DEGs in the H&A were noteworthy because they are known to drive protective functions (*KCNN4*, *TTLL6*, and *EOMES*) (Figure 3b). *KCNN4* downregulation is known to improve tissue protection via decreasing inflammation [56,57,58], *TTLL6* downregulation is known to promote neuron survival via decreasing synaptic damage [59], and *EOMES* downregulation has been shown to promote survival via decreasing cytotoxicity [60,61,62]. This indicates the progressive downregulation of specific genes driving protection and survival from 1 day post-injury to 1 week post-injury in the H&A. *TTLL6* was part of the top 5 downregulated DEGs in the H&A at 1 week post-injury. There were no Intensified upregulated DEGs in either region; all were downregulated DEGs.

For Persistent downregulated DEGs, there were no significant overrepresented GO terms. However, 200 and 437 Persistent downregulated DEGs were present in the FC and H&A, respectively, with 53 shared DEGs between regions (Supplementary Table S3, Tab 7,8). Of the 53, *NPAS1*, *MEX3A*, and *VTN* are noteworthy due to their roles in positively regulating neuronal differentiation [63,64,65]. Thus, their downregulation is consistent with reduced neuronal differentiation. However, the downregulation of another noteworthy microglia-specific gene, *SPHK1*, has been identified to relieve neuronal damage [66]. For the Persistent upregulated DEGs in both regions, the top GO terms were related to tau aggregation (*positive regulation of tau protein kinase activity*), neuroprotection (*autophagy*), transport, synaptic signaling, and neurodevelopment (Figure 3c, Supplementary Table S3, Tab 9). Interestingly, the H&A displayed some distinct neurodegenerative (*regulation of neuron death*) and neuroprotective (*amyloid-beta clearance*) GO terms that were not observed in the FC (Figure 3c, Supplementary Table S3, Tab 10). Collectively, our data suggest an Early and Transient initiation of neuronal distress processes at 1 day post-injury, together with an Intensified downregulation of DEGs to drive neuroprotection, and a Persistent up- and downregulation of both neuroprotective and neurodegenerative DEGs in both regions.

### 2.3. Homeostatic, Synaptic, and Neurodevelopment Genes Were Increased, While Immune and Cell Cycle Genes Were Decreased, at 1 Week After Injury

**Figure 4:**
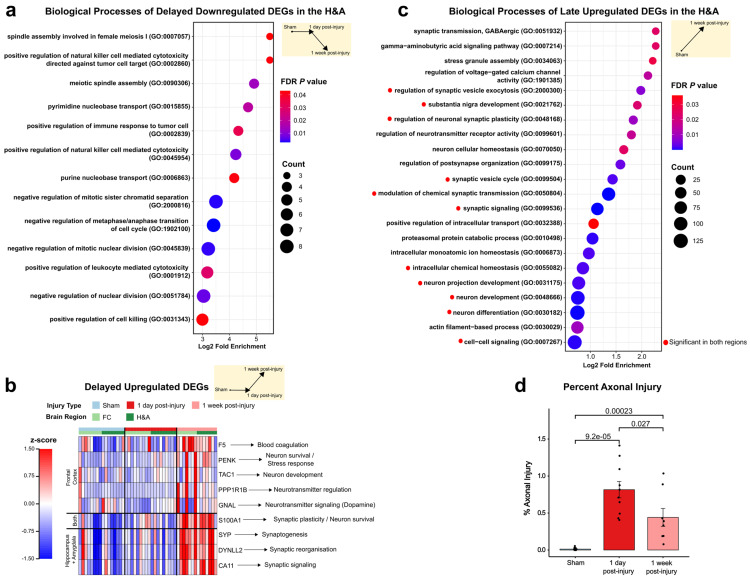
Synaptic, survival, and developmental genes are increased later at 1 week post-injury. (**a**) Top GO terms for the delayed downregulated DEGs in the H&A (FDR ≤ 0.05). Red to blue indicates high to low FDR *p*-values. Count is the number of DEGs making up each GO term. (**b**) Delayed upregulated DEGs. Each row represents a DEG, and each column represents an animal subject (rows were z-scored). (**c**) Top GO terms for the Late upregulated DEGs in the H&A (FDR ≤ 0.05). Red circles indicate those significant in the FC and H&A. Red to blue indicates high to low FDR *p*-values. Count is the number of DEGs making up each GO term. (**d**) Percent axonal injury for Sham and injured groups. Significance was achieved using the Wilcox test, with the Bonferroni adjustment (*p* ≤ 0.05). Data are shown as the mean ± standard error. DEGs, differentially expressed genes. FDR, false discovery rate. FC, frontal cortex. H&A, hippocampus + amygdala.

We next analyzed the functions of DEGs at 1 week post-injury (i.e., Delayed and Late). Within each temporal pattern, we will focus on down- followed by upregulated DEGs. Within the Delayed downregulated DEG group, we identified 20 and 292 DEGs in the FC and H&A, respectively, with 9 DEGs shared between regions. Of the nine shared DEGs, some were known for their roles in immune function or cell cycle. *SHCBP1* is involved in T cell development and cytokinesis during mitosis and meiosis [67], *KIF15* is involved in spindle assembly during mitosis [68], and *ASB9* is known to regulate genes associated with cell cycle progression [69]. *SHCBP1* was one of the top 5 downregulated DEGs in the FC at 1 week post-injury. In the H&A only, PANTHER revealed overrepresented GO terms for the Delayed downregulated DEGs. Similarly, the top GO terms for the Delayed downregulated DEGs in the H&A were related to the cell cycle and immune response (Figure 4a, Supplementary Table S4, Tab 2).

Among the Delayed upregulated DEGs, PANTHER revealed no overrepresented GO terms in either region. The only DEG shared in both regions, *S100A1,* is known for its role in cell survival and neurite outgrowth [70,71] (Figure 4b). Four of the five Delayed upregulated DEGs in the FC only, i.e., *PENK*, *TAC1*, *PPP1R1B*, and *GNAL,* are known for their roles in synaptic signaling and development in striatal neurons (medium-spiny neurons and interneurons) [72,73,74,75] (Figure 4b, Supplementary Table S4, Tab 3). *PENK and PPP1R1B* were part of the top 5 upregulated DEGs in the FC at 1 week post-injury. Likewise, all three Delayed upregulated DEGs in the H&A only, *SYP*, *DYNLL2*, and *CA11,* are involved in synaptogenesis (*SYP*), synaptic plasticity (*SYP*, *DYNLL2*, *CA11*), and the recycling of synaptic vesicles (*SYP*) [76,77,78,79,80,81] (Figure 4b, Supplementary Table S4, Tab 4).

The Late downregulated DEGs shared similar functional annotations with the Delayed downregulated group (Figure 4a). These included cell cycle, neurotransmitter, and hormone uptake processes, among the top GO terms observed in the FC (Supplementary Table S4, Tab 5), while a prominent immune response was observed in the H&A (Supplementary Table S4, Tab 6). These results are consistent with a prior report of a reduced cell cycle conferring neuroprotection and reduced immune response following TBI [82]. For the Late upregulated DEGs, the top GO terms in both regions were associated with neuronal homeostasis, transport, neurodevelopment, and synaptic signaling (Figure 4c, Supplementary Table S4, Tab 7). Interestingly, only the H&A had a response to stress processes (*stress granule assembly*) (Figure 4c, Supplementary Table S4, Tab 8). The Delayed and Late upregulated DEGs were related to neuronal function. Importantly, the upregulation of neuronal activity at 1 week post-injury (Delayed and Late) goes hand in hand with the identified upregulation of neuronal-specific DEGs (Figure 2f).

Overall, our results are consistent with a delayed neurorecovery process, which is highlighted by an increase in neuron development, synaptic signaling, and neuronal survival responses, in addition to a decrease in the cell cycle and immune processes at 1 week post-injury.

To understand if the neuronal distress changes at 1 day and neurorecovery processes at 1 week post-injury correlated with tissue pathology, we examined the extent of axonal injury throughout the brains in the Sham and injured subjects via APP staining [83,84]. A Wilcox test identified a significantly larger fraction percent of the brain testing positive for axonal injury at 1 day post-injury and 1 week post-injury compared to Sham. The percent axonal injury in the 1 week post-injury group was significantly lower than the 1 day post-injury group (Figure 4d). Simultaneously, we identified decreases in Transient and Early survival DEGs (Figure 3a) and GO terms at 1 day post-injury, in addition to increases in Persistent neurodegenerative DEGs (Figure 3c). Others have reported that the accumulation of β-amyloid precursor protein (β-APP), which is a marker for axonal injury, is a hallmark of neurodegeneration [85]. Additionally, the significant decrease in percent axonal injury from 1 day to 1 week post-injury was concurrent with the neurorecovery responses that were initiated at 1 week post-injury (i.e., Delayed and Late) (Figure 4a-c). Taken together, the DEGs observed at 1 day and 1 week post-injury are consistent with the temporal alterations in axonal injury.

### 2.4. Emerging Trends: Commonalities Across Region and Time

**Figure 5:**
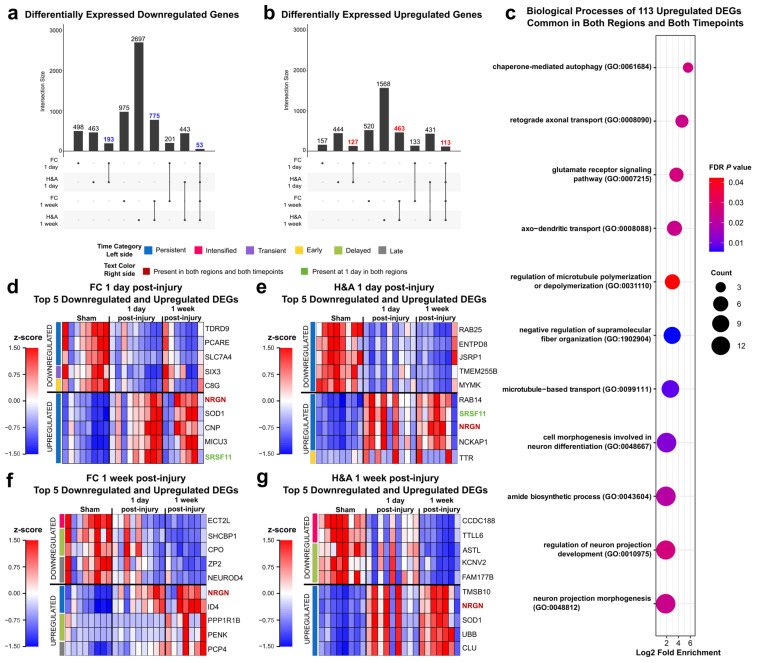
Common and top 5 DEGs in both regions and at both timepoints. (**a**) Upset plot depicting the number of overlapping downregulated DEGs (unadjusted *p* ≤ 0.05, |log2FC| ≥ 1) between groups. (**b**) Upset plot depicting the number of overlapping upregulated DEGs (unadjusted *p* ≤ 0.05, |log2FC| ≥ 1) between groups. (**c**) Top GO terms for the 113 upregulated DEGs in both regions and at both timepoints (FDR ≤ 0.05). Red to blue indicates high to low FDR *p*-values. Count is the number of DEGs making up each GO term. (**d**) Top 5 downregulated and upregulated DEGs in the FC at 1 day post-injury (FDR ≤ 0.05, |log2FC| ≥ 1). (**e**) Top 5 downregulated and upregulated DEGs in the H&A at 1 day post-injury (FDR ≤ 0.05, |log2FC| ≥ 1). (**f**) Top 5 downregulated and upregulated DEGs in the FC at 1 week post-injury (FDR ≤ 0.05, |log2FC| ≥ 1). (**g**) Top 5 downregulated and upregulated DEGs in the H&A at 1 week post-injury (FDR ≤ 0.05, |log2FC| ≥ 1). *NRGN* (bold red) is present in all groups. *SRSF11* (bold green) is present in both regions at 1 day post-injury. Each row represents a DEG, and each column represents an animal subject (rows were z-scored). DEGs, differentially expressed genes. FC, frontal cortex. H&A, hippocampus + amygdala.

After determining the cell affinities and functionalities of all six temporal patterns, we next identified common DEGs across regions and/or timepoints. We focused on three groups of DEGs: (i) significant in both regions at 1 day post-injury, (ii) significant in both regions at 1 week post-injury, and (iii) significant in both regions and at both timepoints. Interestingly, we observed many more DEGs common to both regions at 1 week post-injury (775 downregulated and 463 upregulated) than at 1 day post-injury (193 downregulated and 127 upregulated) (Figure 5a,b). The 193 downregulated DEGs that were common in both regions at 1 day post-injury were associated with a variety of functions related to ROS production, immune infiltration, synaptic transmission, neuron survival, and development (Supplementary Table S5, Tab 1). The 127 upregulated DEGs that were common in both regions at 1 day post-injury had top GO terms associated with tau kinase activity, autophagy, synaptic signaling, and neuron development (Supplementary Table S5, Tab 2). These functions were similar to those observed in the Transient, Early, and Persistent temporal patterns (Figure 3a,c). The 775 downregulated DEGs that were common in both regions at 1 week post-injury had top GO terms associated with transport and cell cycle processes (Supplementary Table S5, Tab 3). The 463 common upregulated DEGs in both regions had top GO terms associated with synaptic signaling, amyloid beta clearance and formation, autophagy, and neuronal transport (Supplementary Table S5, Tab 4). These GO annotations had similar functions to those of the Delayed downregulated and Persistent upregulated temporal patterns (Figure 3c and 4a). Finally, 53 downregulated DEGs and 113 upregulated DEGs were common to both regions and both timepoints, and all were in the Persistent temporal pattern. The 53 downregulated DEGs were involved in both neurodegenerative and neuroprotective functions (Supplementary Table S5, Tab 5). The 113 DEGs were explored further using PANTHER, and the top overrepresented GO terms were related to neurorecovery processes like autophagy, transport, and neuron development (Figure 5c, Supplementary Table S5, Tab 6).

### 2.5. Emerging Trends: Largest Fold Changes

Next, we asked which DEGs showed the greatest changes in response to injury, compared to Sham (i.e., FDR ≤ 0.05 and largest log2FC). We identified the top 5 downregulated and top 5 upregulated DEGs in each region and at each timepoint. First, by examining the log2FC, we observed higher log2FC ranges in the H&A compared to the FC (Table 1).

Focusing on the top 5 DEGs in each region and timepoint, we noted that at 1 day post-injury, there was one Early and one Transient DEG. Specifically, *C8G* was the only Early downregulated (yellow bar on left) DEG in the FC, while *SIX3,* which is involved in neuron survival, production, and growth [45,46,47], was the only Transient (purple bar on left) downregulated DEG in the FC (Figure 5d). Similarly, in the H&A, *TTR*, which is known to assist in amyloid beta aggregation inhibition [86], was the only Early upregulated gene among the top 5 DEGs (Figure 5e). At 1 day post-injury, most of the top 5 down- and upregulated DEGs in the FC (Figure 5d) and H&A (Figure 5e) were Persistent (blue bar on left). At 1 week post-injury, temporal patterns were more heterogeneous in the FC (Figure 5f), but Persistent DEGs were mainly in the H&A (Figure 5g). The majority of the top 5 Persistent upregulated DEGs at 1 day and 1 week post-injury were involved in neuron growth (*CNP*, *NCKAP1*) [87,88], amyloid beta clearance (*CLU*, *TTR*) [86,89], neuroprotection (*CNP*) [90], synaptic plasticity (*CNP, MICU3*) [91,92], and BBB integrity (*CNP*) [93].

Interestingly, at 1 week post-injury, the three Intensified DEGs (pink bar on left) were all downregulated in both regions (Figure 5f,g). *ECT2L* was Intensified downregulated in the FC and is known to be involved in the regulation of Rho protein signal transduction [94]. *CCDC188* and *TTLL6* were Intensified downregulated in the H&A. Of these, *TTLL6* is highly expressed in astrocytes [95] and known to promote synaptic damage [59]. Interestingly, its expression was further downregulated at 1 week post-injury, suggesting decreased neuronal damage at this timepoint.

*SCHBP1* and *CPO* were the two Delayed downregulated DEGs (green bar on left) in the FC and are known for their roles in positively regulating cell cycle progression (*SCHBP1*) [96] and C-terminal amino acid cleavage (*CPO*) [97] (Figure 5f). *ASTL*, *KCNV2,* and *FAM177B* were the three Delayed downregulated DEGs in the H&A (Figure 5g). *KCNV2* is known as a marker for severe epilepsy [98] and *FAM177B* is known to be exclusively expressed in microglia [99]. The two Delayed upregulated DEGs in the FC were *PPP1R1B* and *PENK,* which have been previously highlighted for their roles in synaptic signaling and development in striatal neurons (medium-spiny neurons and interneurons) [73,74,75] (Figure 5f). Thus, the top 5 Delayed down- and upregulated DEGs were involved in both neuronal and immune processes.

From our top 5 lists, Late DEGs (gray bar on left) were only observed in the FC (Figure 5f). *ZP2* and *NEUROD4* were the two Late downregulated DEGs, while *PCP4* was the only Late upregulated DEG in the FC. These Late DEGs were involved in neuronal processes. The downregulated DEG *NEUROD4* is involved in neuron differentiation [100]. Similarly, the upregulated DEG *PCP4* is involved in neurite outgrowth, neurotransmitter release, and Purkinje cell excitability [101,102].

Finally, we identified DEGs that were common in both regions at 1 day post-injury (1 DEG, green text) and in both regions and at both timepoints (1 DEG, red text) (Figure 5d–g). These two DEGs with regional and/or temporal commonality were Persistent. The only top 5 DEG that was common to both regions at 1 day post-injury, *SRSF11,* is known to improve cognitive function by controlling the activation of the JNK pathway [103] (Figure 5d,e). The top 5 DEG common across regions and timepoints, *NRGN,* is associated with synaptic functions. Its overexpression is known to enhance synaptic strength and lowers the threshold for long-term potentiation (LTP) induction (Figure 5d–g) [104,105,106]. It has also been involved in the rearrangement of the cytoskeletal structures of neurons [107]. Additionally, it has been identified as a serum biomarker in patients following acute TBI [108]. In total, the common top 5 DEGs (colored text) were associated with cognitive improvement and neurorecovery through regulating neuronal responses.

### 2.6. Cyclosporine A (CSA) Treatment Accelerates Neurorecovery Processes

We previously reported that cyclosporine A (CsA, 20 mg/kg) administered 6 h post-injury decreased brain lesion volume and mitigated alterations in mitochondrial health in the cortex and hippocampal regions at 1 day post-injury [24]. We thus hypothesized that CsA would promote changes in genes associated with neurorecovery. To test this, we collected frontal cortex (FC) and hippocampus + amygdala (H&A) RNA-seq data from injured CsA-treated animals and compared the results to the Sham, untreated groups at 1 day and 1 week post-injury. We then evaluated two primary effects of CsA on the DEGs identified in the 1 day and 1 week post-injury groups:

1. **Dampened DEGs:** These were CsA-responsive DEGs that normalized to Sham levels upon CsA treatment; DEGs that were ameliorated (not significant in treated vs Sham) in injured subjects after 1 day of CsA treatment to levels similar to uninjured Shams; Transient, Early, Persistent, and Intensified DEGs whose expressions became similar to Sham following CsA treatment (Figure 2).
2. **Accelerated DEGs:** These were CsA-fast-responsive DEGs that changed to levels similar to the untreated 1 week post-injury group within 1 day of CsA treatment; DEGs in injured subjects that were assessed just 1 day after CsA treatment that were increased or decreased to levels similar to untreated injured subjects at a 1 week post-injury timepoint; Late or Delayed DEGs now altered at 1 day following CsA treatment (Figure 2).

**Figure 6:**
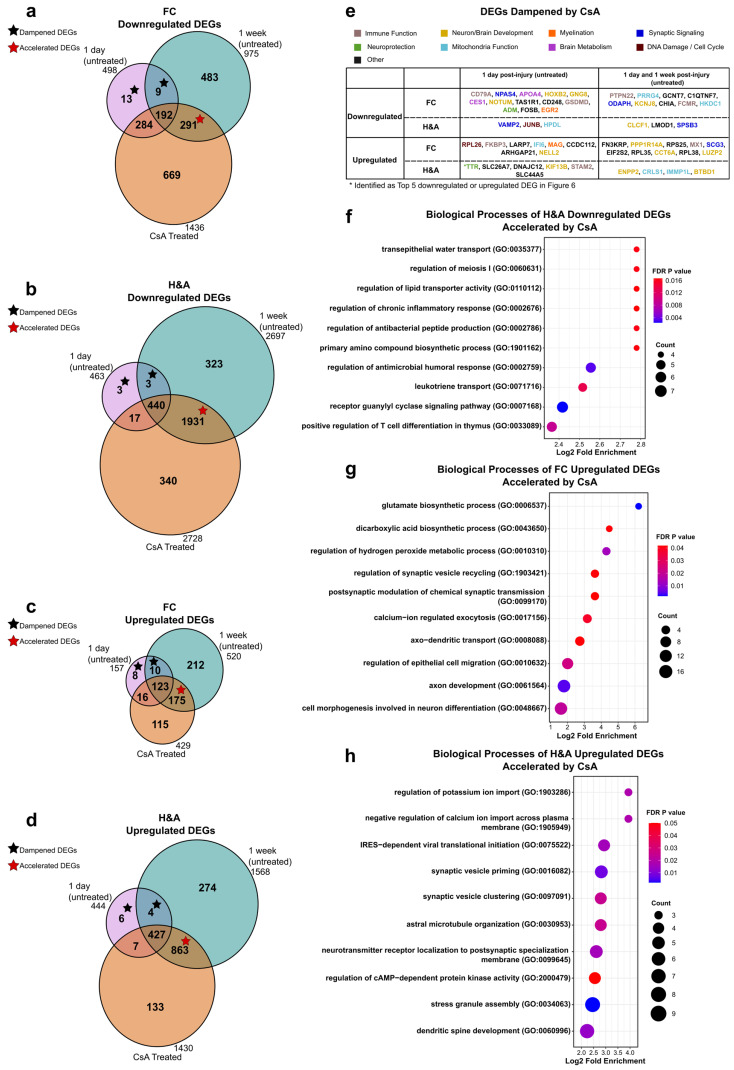
Cyclosporine A accelerates neurorecovery DEGs. Venn diagrams highlighting dampened (black stars) and accelerated DEGs (red stars). The pink circle represents DEGs in the 1 day post-injury vs. Sham comparison, the green circle represents DEGs in the 1 week post-injury vs. Sham comparison, and the orange circle represents DEGs in the CsA-treated vs Sham comparison. Dampened DEGs are in the 1-day-post-injury-only and 1-day-post-injury + 1-week-post-injury sections. Accelerated DEGs are in the 1-week-post-injury + CsA-treated sections for the (**a**) FC downregulated, (**b**) H&A downregulated, (**c**) FC upregulated, and (**d**) H&A upregulated DEGs. (**e**) Functions of dampened DEGs. (**f**) Top GO terms of accelerated downregulated DEGs in the H&A (FDR ≤ 0.05). Red to blue indicates high to low FDR *p*-values. Count is the number of DEGs making up each GO term. (**g**) Top GO terms of accelerated upregulated DEGs in the FC (FDR ≤ 0.05). Red to blue indicates high to low FDR *p*-values. Count is the number of DEGs making up each GO term. (**h**) Top GO terms of accelerated upregulated DEGs in the H&A (FDR ≤ 0.05). Red to blue indicates high to low FDR *p*-values. Count is the number of DEGs making up each GO term. FDR, false discovery rate. FC, frontal cortex. H&A, hippocampus + amygdala. CsA, cyclosporine A.

We found that CsA treatment at 1 day post-injury accelerated many more DEGs (red stars) than were dampened (black stars, Figure 6a–d). Dampened DEGs were those present in the 1-day-only and 1-day-post-injury + 1-week-post-injury intersections (black stars). Accelerated DEGs were those present in the 1-week-post-injury + CsA-treated intersections (red star).

There were few dampened DEGs in the FC (22 down, 18 up) and the H&A (6 down, 10 up), resulting in no significant GO terms (Figure 6e, Supplementary Table S6, Tab 1–4). The few DEGs dampened by CsA were mostly associated with neuron development and mitochondria functions (Figure 6e). Of the 16 dampened DEGs in the H&A, we highlighted 2 interesting DEGs, *VAMP2* and *TTR*. The synaptic signaling gene *VAMP2* was the only Transient downregulated DEG in the H&A (Figure 3a), while *TTR* was the only top 5 Early upregulated DEG in the H&A at 1 day post-injury (Figure 5e). *TTR* plays a neuroprotective role in amyloid beta aggregation inhibition and protection from brain trauma [86,109], illustrating that CsA can also modulate neuroprotective genes by bringing them back to their levels of homeostasis.

With respect to the accelerated DEGs, more DEGs were accelerated in the H&A compared to the FC. Among the downregulated DEGs, there were no significant GO terms in the FC (Supplementary Table S6, Tab 5). However, one of the downregulated DEGs accelerated by CsA in the FC, *SCHBP1*, was part of the top 5 downregulated DEGs in the FC at 1 week post-injury (Figure 5f). Its downregulation is known to positively regulate the inhibition of cell proliferation and cell cycle progression by inhibiting cyclin D1 and CDK4 [96]. Two other interesting downregulated DEGs accelerated by CsA to drive neurorecovery in the FC were the pro-inflammatory genes *IL20RA* and *FAM124B*. *FAM124B* is involved in the pathology of neurodevelopmental disorders [110]. In the H&A, GO terms from accelerated downregulated DEGs were associated with blood–brain barrier transport (*transepithelial water transport*), cell cycle, lipid transport, nitric oxide signaling (*guanylyl cyclase signaling pathway*), and immune responses (Figure 6f, Supplementary Table S6, Tab 6), reflecting that CsA treatment accelerated downregulated stress and immune responses.

Among the upregulated accelerated DEGs, in the FC, significant GO terms were related to synaptic signaling, transport, and axon development (Figure 6g, Supplementary Table S6, Tab 7). Similarly, in the H&A, the top GO terms were related to synaptic signaling and brain development, in addition to responses to stress processes (*stress granule assembly*) (Figure 6h, Supplementary Table S6, Tab 8). These processes suggest that CsA treatment accelerates the upregulation of synaptic signaling and development processes in both regions, as well as the response to stress processes in the H&A. In total, these data suggest that CsA treatment primarily accelerates neuronal recovery processes, rather than dampening neuronal distress processes.

### 2.7. WGCNA Highlights Modules of Genes Dampened or Accelerated by CsA Treatment

**Figure 7:**
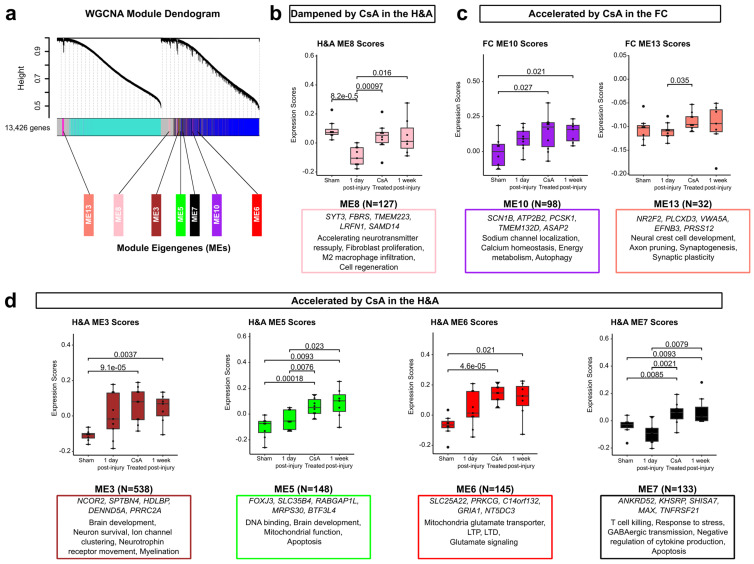
Functional modules dampened or accelerated by CsA treatment. (**a**) Hierarchical clustering tree of 13,426 genes. Each gene cluster (module) has a distinct color representing co-expressed genes. A total of 13 modules were identified. Modules highlighted are those dampened or accelerated in either region. (**b**) Expression scores of the dampened module, ME8 (pink), in the H&A. The box includes its top 5 hub genes and their functions. (**c**) Expression scores of the accelerated modules, ME10 (purple) and ME13 (salmon), in the FC. The boxes include their top 5 hub genes and their functions. (**d**) Expression scores of the accelerated modules, ME3 (brown), ME5 (green), ME6 (red), and ME7(black), in the H&A. The boxes include their top 5 hub genes and their functions. All stats, Wilcox test, Bonferroni-adjusted *p* ≤ 0.05). Data are shown as the mean ± standard error. FC, frontal cortex. H&A, hippocampus + amygdala. CsA, cyclosporine A. ME, module eigengene.

To gain holistic insight into how genes change together with CsA treatment, we examined modules of co-expressed genes using weighted gene co-expression network analysis (WGCNA). WGCNA was conducted on all 13,426 genes shown in Figure 1, resulting in a total of 13 functional modules of highly co-expressed genes, as depicted by the module clustering dendrogram (Figure 7a; Supplementary Table S7). Next, we asked if we could identify modules accelerated or dampened by CsA. Although no modules were dampened by CsA in the FC, ME8 was dampened by CsA in the H&A (Figure 7b). In contrast, ME10 and ME13 were accelerated by CsA in the FC (Figure 7c), while ME3, ME5, ME6, and ME7 were accelerated by CsA in the H&A (Figure 7d).

Examining ME8 in more detail, regarding the downregulated module dampened by CsA in the H&A (Figure 7b), its top 5 hub genes are more highly expressed in astrocytes and neurons [95] and are involved in synaptic transmission (*SYT3*) [111], fibroblast proliferation (*FBRS*) [112,113,114], cytochrome c oxidase biogenesis (*TMEM223*) [115], promoting M2 macrophage infiltration (*LRFN1*) [116], and cell regeneration and survival (*SAMD14*) [117]. These hub genes are consistent with CsA restoring the downregulation of neuronal protective and regenerative processes in the H&A at 1 day post-injury.

Examining MEs 10 and 13, which were accelerated in the FC by CsA, most of their genes were neuron-specific (Figure 7c; Supplementary Figure S2). The top 5 hub genes in ME10 have roles in sodium channel localization (*SCN1B*) [118], calcium homeostasis (*ATP2B2*) [119], energy metabolism (*PCSK1*) [120], cell adhesion (*TMEM132D*) [121], vesicular transport (*ASAP2*) [122], and autophagy (*ASAP2*) [122]. The top 5 hub genes in ME13 are highly expressed in neurons [95] and have roles in neural crest cell development (*NR2F2*) [123], axon pruning (*EFNB3*) [124], synaptogenesis (*EFNB3*) [125], synaptic plasticity (*EFNB3*) [125], and the development of the brain (*PRSS12*) [126]. Thus, in the FC, CsA accelerates hub genes associated with neuronal signaling, development, homeostasis, and protection.

Examining MEs 3, 5, 6, and 7, accelerated in the H&A by CsA (Figure 7d), we found that the genes in ME3, ME6, and ME7 were mostly astrocytes and neuron-specific, while those in ME5 were mostly microglia- and neuron-specific (Supplementary Figure S2). The top 5 hub genes in ME3 are known for their roles in brain development (*NCOR2*) [127], neuron survival (*NCOR2*) [128], voltage-gated sodium channel clustering (*SPTBN4*) [129], cholesterol transport (*HDLBP*) [130], neurotrophin receptor movement (*DENND5A*) [131], and myelination (*PRRC2A*) [132]. The top 5 hub genes in ME5 are known to be highly expressed in neurons [95], and some are involved in DNA binding during development (*FOXJ3*) [133], brain development (*RABGAP1L*) [134], mitochondrial function (*MRPS30*) [135], and apoptosis (*MRPS30*) [135]. The top 5 hub genes in ME6 are known for their roles in driving glutamate transport (*SLC25A22*) [136], long-term potentiation (*PRKCG*) [137], long-term depression (*PRKCG*) [137], and glutamate signaling (*GRIA1*) [138]. The top 5 hub genes in ME7 have roles in T cell killing (*ANKRD52*) [139], negative regulation of cytokine production (*KHSRP*) [140], response to stress (*ANKRD52*) [141], GABAergic transmission (*SHISA7*) [142], apoptosis (*MAX*) [143], and apoptosis with JNK and NF-κB pathway activation (*TNFRSF21*) [144]. Therefore, in the H&A, CsA accelerates hub genes associated with neuronal signaling, development, mitochondrial function, and reduced immune signaling. Collectively, these modules define groups of co-expressed genes dampened or accelerated by CsA. These modules are associated with increased neuronal signaling, development, protection, and repair, in addition to decreased immune signaling, further highlighting the potential role of CsA in hastening neurorecovery post-injury.

**Figure 8:**
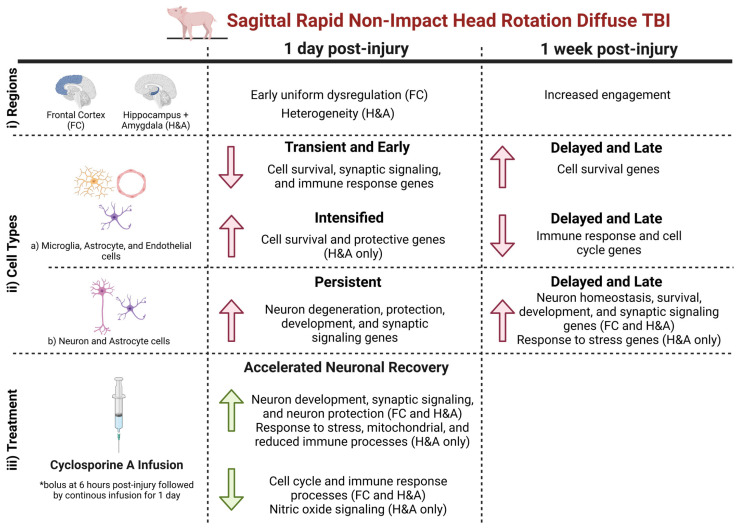
Results summary. Created in BioRender. Wood, L. (2025) https://BioRender.com/2oqfrhu (accessed on 4 April 2025).

In summary, following a sagittal RNR, by focusing on (i) region, we observed that the FC displayed early uniform dysregulation, while the H&A displayed subject-to-subject heterogeneity at 1 day post-injury. Additionally, at 1 week post-injury, there were an increased number of DEGs in both regions. By focusing on (ii) cell type-specific DEGs, we observed that (a) microglia-, astrocyte-, and endothelia-specific DEGs, which were classified as Early and Transient, were associated with a decrease in cell survival, synaptic signaling, and immune responses in both regions, while those which were Intensified were associated with an increase in cell survival and protective processes in the H&A. Delayed and Late DEGs were associated with an increase in cell survival processes and a decrease in immune and cell cycle processes in both regions. In contrast, (b) neuron- and astrocyte-specific DEGs that were designated as Persistent DEGs were associated with an increase in neuron degenerative and protective processes in both regions, while those that were Delayed and Late DEGs were associated with an increase in neuron homeostasis, survival, development, and synaptic signaling processes in both regions, and an increased response to stress in the H&A. (iii) In both regions, post-injury cyclosporine A treatment triggered an accelerated neuronal recovery process by accelerating genes associated with an increase in neuron development, synaptic signaling, and protection, and a decrease in cell cycle and immune response processes. Within the H&A, it also accelerated DEGs associated with the response to stress, mitochondria function, reduced immune processes, and decreased nitric oxide signaling (Figure 8).

## 3. Discussion

In this study, we examined the changes in gene expression in the frontal cortex (FC) and hippocampus + amygdala (H&A) regions of a pediatric swine model of mTBI at 1 day and 1 week post-injury and following cyclosporine A (CsA) treatment at 1 day post-injury. By conducting bulk RNA sequencing, we identified cell type-specific up- and downregulated DEGs associated with neuronal distress at 1 day, neurorecovery at 1 week, and an acceleration of neurorecovery-associated genes at 1 day post-injury with CsA treatment. We employed a piglet model of mTBI in this work because of the established parallels between pig and human brains in neurological anatomy and development [145,146,147,148,149]. Pediatric TBI affects 475,000 children per year, with a paucity of effective therapies [150].

Multiple studies have reported that children and adults respond differently to TBI [151,152], underscoring the importance of studying the immature brain. Additionally, most of the adult and pediatric TBI studies have utilized rodents, which are popular animal models for implementing the effects of injury severity, age, gender, and time post-injury in studying gene expression in various TBI models. Nevertheless, rodent brains are smaller, lissenphalic, and have less white matter tissue [153,154,155] compared to human brains [156,157]. Moreover, the focal impact model in rodents fails to capture the head motion and brain structure kinematics interplay that frequently characterize the biomechanics of diffuse brain injury observed in humans [157,158,159,160,161]. Due to the complexity of human TBI, some studies have also raised the possibility of differences in responses to trauma, like glial injury responses and repair in rodents compared to humans [155,162]. Alternatively, pigs possess gyrencephalic brains that are similar in white and gray matter distribution compared to human brains [145,146]. Their similar and well-formed gyri and sulci also enable pigs to better recapitulate pathologies of white matter injuries identified in humans [153,157,158,163]. The rapid postnatal development of the neonatal pig has well-characterized similarities with a human newborn infant in terms of myelination, composition, and electrical activity [147,148,149,164,165,166]. Neonatal piglets, piglets between 1 and 5 days old, piglets between 3 and 4 weeks old, and 4–5-month-old piglets are comparable to newborn humans, infants, toddlers/preschoolers, and prepubescents, respectively [167,168,169,170,171,172]. Additionally, pigs have been identified to share similar responses to trauma compared to humans. For example, following a rotational injury using a pneumatic “HYGE, Inc.” device, Cullen et al., 2016 [173] identified similarities in trauma-induced axonal undulations in the brain tissue of a pig and a human post-TBI. These similarities in development, morphology, and pathology between a piglet and human brain provide advantages in modeling age-specific responses to brain trauma in order to study pediatric mTBI [167]. Our findings inform a gap in the understanding of pediatric mTBI, by defining key molecular changes associated with region, elapsed time, and CsA treatment following rapid head rotations.

### 3.1. Early Alteration of Genes in the Frontal Cortex (FC) at 1 Day Post-Injury

Our analysis revealed an early and uniform dysregulation of genes in the FC at 1 day post-injury. Interestingly, we also observed a higher number of Early and Transient DEGs in the FC compared to the H&A. Resolved after 1 day post-injury, these Early and Transient changes were associated with decreased cell survival, cell adhesion, immune signaling, and synaptic signaling processes, which could lead to neurodegeneration. Additionally, we observed an Early downregulation of microglia-, astrocyte-, and endothelia-specific DEGs in the FC. The frontal lobe is known to be vulnerable and susceptible to TBI, and early injury or degeneration in this region has been reported. A study in TBI piglets noted axonal injury in the frontal lobe as early as 6 h post-injury [43]. Additionally, another study in TBI rats observed early decreases in synaptic integrity and microglial and astrocytic cells in the pre-frontal cortex at 1 month post-injury, which were all resolved by 3 months post-injury [174]. Interestingly, although some changes returned to Sham levels by 3 months post-injury, the researchers still found evident persistent pre-frontal cortex impairments (i.e., depression). Similarly, our study also identified that Persistent DEGs associated with neurodegeneration were upregulated in the FC. These neurodegenerative DEGs could be associated with FC-related impairments. Overall, consistent with prior studies, our study shows that the FC is affected early post-injury compared to the H&A and can sustain persistent alterations for up to 1 week post-injury.

### 3.2. Increased and Uniform Engagement of Hippocampus + Amygdala (H&A) at 1 Week Post-Injury Following Initial Heterogeneity

In contrast to the FC, we report subject-to-subject heterogeneity in gene expression in the H&A at 1 day post-injury. The hippocampus location is known to be more protected from injury in human and pig brains because it lies deep inside the temporal lobe and is positioned more ventrally [175,176]. However, by 1 week post-injury, the H&A became more uniform, with a delayed increase in the number of DEGs compared to 1 day post-injury, and more Delayed and Late DEGs compared to the FC. Studies have observed a delayed increase in hippocampus damage [177,178,179] and hippocampus recovery [180,181,182,183] compared to other regions following brain injury. A study conducted in patients after cardiorespiratory arrest observed that compared to other regions, there were delayed increases in hippocampal damage with time post-arrest [184]. However, in our piglet study, we observed that the delayed engagement of the H&A at 1 week post-injury was associated with the emergence or intensification of neuronal recovery processes. Consistent with our results, an mTBI study in rat pups also observed spontaneous recovery over the first week post-injury, although the hippocampus was identified to be vulnerable to TBI [185]. Two other studies, one in neonatal rats and the other in juvenile rats, observed a delayed increase in cell proliferation in only the hippocampus at 5 days and 2 days post-injury, respectively [22,181]. The study at 2 days post-injury also noted that neurogenesis in the hippocampus was far greater in the juvenile rats compared to adults [22]. This trend in pediatric-aged animal models of hippocampal cell proliferation and recovery, rather than damage, raises the question as to whether the delayed engagement of the hippocampus later in injury is age-related and specifically drives recovery in younger-aged animal models. Additionally, we observed that Intensified downregulated DEGs in the H&A were also involved in positively regulating neuronal survival and protection. Overall, consistent with published pediatric studies, we observed delayed recovery in the H&A associated with a post-injury intensification and delayed emergence of neuronal protective and recovery DEGs.

### 3.3. Increased Neuron Degenerative and Protective DEGs After 1 Day

Focusing on the DEGs observed in both regions at 1 day post-injury, we observed Transient or Early DEGs associated with decreases in cell survival, synaptic signaling, and immune response processes. Those classified as Persistent included those indicating an upregulation of DEGs associated with neuron degeneration, development, and protection. These concurrent protective and degenerative responses observed from the Persistent DEGs underscore the complexity of the brain’s response to TBI. Although many studies, especially in adult TBI, focus on the persistent degenerative cascades [186,187], some have also identified this similar interplay between both neurodegenerative and neurorestorative/neuroprotective events as early as hours or days post-injury [188,189]. Interestingly, most Persistent DEGs from our top 5 (i.e., largest log2FC fold change) up- and downregulated list (Figure 5) were associated with neuroprotection, especially those common in both regions and at both timepoints. These high-fold neuroprotective changes may reflect a balance tipped toward driving restoration and protection post-injury, consistent with pediatric studies that have reported recovery and neurogenesis post-injury, specifically in TBI [22,190]. Overall, consistent with other studies, we observed an interplay between Persistent neurodegenerative and neuroprotective processes after 1 day post-injury, with large fold changes in neuroprotective processes.

### 3.4. Delayed Emergence of Neuronal Recovery Processes Until 1 Week

Interestingly, at 1 week post-injury, many of the upregulated Delayed and Late DEGs present in both regions were astrocyte- and neuron-specific, and known for their roles in driving neuronal growth, signaling, homeostasis, survival, and protection. Similarly, studies in TBI and stroke have also noted increases in neuronal growth and recovery around 1 week to months post-TBI [191,192,193,194]. Also, crosstalk between astrocytes and neurons has been identified to actively control brain homeostasis, neuronal metabolism, and synaptic connectivity [195,196], which align with the functions of these Delayed and Late upregulated DEGs. Overall, in our study, DEGs specific to neurons and astrocytes were significantly upregulated to provide restorative and survival functions post-injury.

Simultaneously, we observed a Delayed and Late downregulation of glial- and endothelial-specific DEGs in both regions at 1 week post-injury. These downregulated DEGs were associated with inflammatory/immune and cell cycle processes. Previously, a decrease or inhibition of the cell cycle was reported to provide neuroprotection and reduced immune responses like glial proliferation post-injury [82]. Overall, it was interesting to observe a combined decrease in cell cycle and immune processes at 1 week post-injury.

### 3.5. Cyclosporine A Accelerates the Expression of Genes Associated with Improved Outcome

Our study is the first to examine transcriptional profiles modulated by CsA treatment following diffuse mTBI in a pediatric model. CsA has been previously described as a restorative treatment that improves, inhibits, or decreases impaired molecular processes to drive improved outcomes. Interestingly, we found that CsA accelerated the up- and downregulation of DEGs associated with driving neuronal recovery processes post-injury, rather than dampening/restoring DEGs associated with neuronal distress at 1 day post-injury. The accelerated DEGs were associated with increased neuronal development and synaptic signaling and decreased stress and immune signaling. Although this was observed in both regions, there were more DEGs accelerated in the H&A. Previous studies have highlighted the roles of CsA in improving impaired long-term potentiation (LTP) [197], improving damaged synaptic mitochondria [198,199], reducing glial activation and pro-inflammation [200,201], reducing lesion volume, and improving mitochondria dysfunction [23,24]. Our study extends our understanding by showing that CsA accelerates neuronal recovery genes in a pediatric mTBI model.

### 3.6. Limitations and Future Work

We had several limitations in this study. First, this work was enabled by using FFPE brain tissues. Although RNA sequencing of FFPE tissues typically suffers from lower RNA yield and quality [202], we removed excess paraffin from each tissue slide to obtain higher-yield RNA during isolation. Second, we utilized a single, well-characterized rapid sagittal head rotation without impact. In the field, head rotation direction may vary, and TBIs may occur with and without impact. The goal of this study was to focus on the effect of elapsed time, injury, and CsA treatment without having the covariates of other injury types and head kinematics because studies have demonstrated that different brain injuries have different mechanisms and physiological characterizations [203]. Additionally, we utilized this model because a sagittal rotation is known to capture brain injuries observed in children [204] and also produces worse outcomes and greater axonal injury compared to other directions [43]. The third limitation is the use of only female piglets. Women are known to experience worse overall TBI outcomes [205,206,207,208]. Because sex hormones can lead to faster recovery post-injury [209,210,211], we limited our study to prepubertal female piglets. The fourth limitation is the use of unadjusted *p*-values rather than FDR-adjusted *p*-values to define DEGs. We focused on being consistent with prior studies and wanted to provide a reasonably permissive *p*-value threshold. We also effectively filtered DEGs based on a |log2FC| ≥ 1 and intersections within temporal patterns and treatment groups. Additionally, we have included volcano plots of the DEGs with an FDR ≤ 0.05, |log2FC| ≥ 1 in Supplementary Figure S1. In Supplementary Table S8, we have also listed all the DEGs in our study and included their FDR-adjusted *p*-values to help researchers interested in furthering our work. Future studies are required to establish the mechanisms of CsA neuroprotection and to define relevant CsA parameters for clinical translation. Understanding DEGs accelerated by CsA will enable follow-on causal studies of which genes would be most beneficial as therapeutics. Additionally, future work will also focus on other rotational directions, including focal cortical injury models, observe gene expression changes for a longer period, and incorporate both sexes and a broader age span.

## 4. Materials and Methods

### 4.1. Animals

Female Yorkshire swine (*Sus scrofa*) weighing 8.8 ± 1.32 kg (4-week-old) (Table 2) were housed in groups of 2–3 in a housing pen with adequate space to allow socialization and subjected to a 12 h light–dark cycle.

### 4.2. mTBI Model and Surgical Procedures

**Figure 9:**
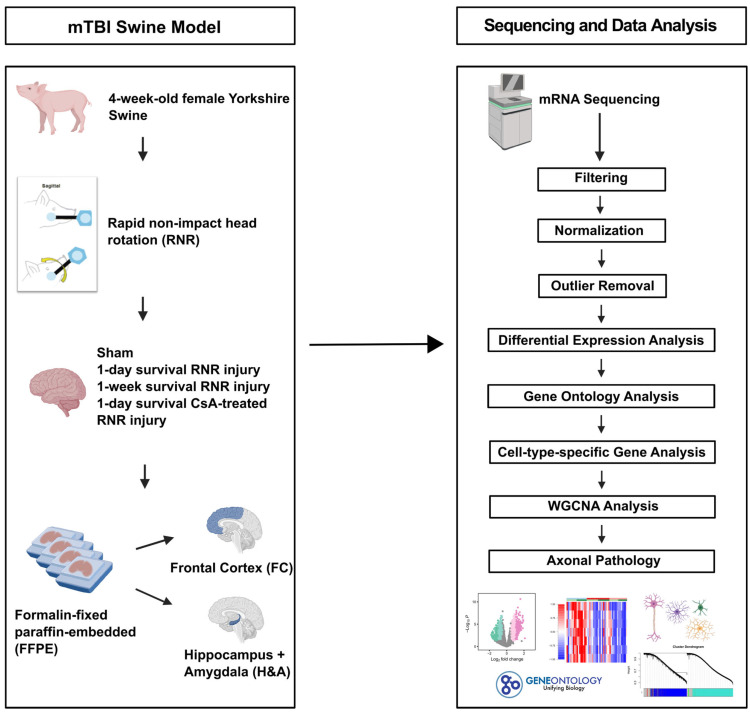
Schematic of experimental design.

A rapid non-impact head rotation (RNR) model in the sagittal plane was utilized for this study. Animals were divided into four experimental groups. (i) Sham: anesthesia-only Sham (n = 11) and Naïve (n = 7), (ii) injured 1-day survival (n = 10), (iii) injured 1-week survival: 6 days survival (n=6) and 8 days survival (n = 9), and (iv) Injured with 1 day of cyclosporine A (CsA) treatment (n = 10). The schematic of the experimental design is shown in Figure 9. The injured CsA-treated animals received a 20mg/kg/day dose of CsA intravenously via an ambulatory wireless programmable infusion pump, delivering a loading dose at 6 h post-injury, followed by continuous infusion for 1 day, thereby making up 30 h of survival post-injury, as described in Margulies et al., 2015 [24]. Both anesthesia-only Sham (2 anesthesia episodes) and Naïve (anesthesia at sacrifice only) animals were combined and designated as the Sham group. Animals were randomized to their study groups.

Prior to injury, all experimental groups were sedated with ketamine/xylazine (20/2 mg/kg) via intramuscular (IM) injection, then subsequently anesthetized with 1–4% isoflurane via a snout mask. Following anesthesia and toe pinch reflex test, animals were intubated and mechanically ventilated while maintained under anesthesia with 2–3% isoflurane. Injured animals were secured on a table, and body temperatures were monitored and maintained at 37°C using a rectal probe and a heating pad. Rapid non-impact head rotations (RNR) were delivered using a pneumatic HYGE device (HYGE, Inc., Kittanning, PA, USA) [173]. Animals’ snouts were secured on a padded snout clamp and rotated 60–70° in the sagittal plane at an average target rotational velocity of 124.6 ± 2.59 rad/s and rotational acceleration of 49.5 ± 11.2 krad/s^2^ (mean ± standard deviation (SD)) (Table 2).

Following head rotations, injured animals survived for 1 day, 30 h (CsA-treated group), and 1 week, then euthanized by sodium pentobarbital overdose. Naïve and Sham animals were sacrificed at 1–8 days, following anesthesia administration (Sham animals only). In this retrospective cohort of convenience, all animals’ brains were perfused, harvested, formalin-fixed, cut into 3mm thick coronal sections, and paraffin-embedded (FFPE), and the blocks were stored in a dry cool cabinet for 7–14 years. We utilized this cohort from our tissue bank of FFPE specimens to facilitate comparison with human studies that also employ postmortem FFPE tissue. Importantly, FFPE tissue can provide reliable gene expression data compared to frozen tissue given proper extraction and quality control [212,213].

### 4.3. mRNA Sequencing Protocol

#### 4.3.1. Total RNA Isolation and Quality Assessment

The standard FFPE tissue slicing and transfer protocol was optimized to maximize RNA yield. Frontal cortex (FC) and hippocampus + amygdala (H&A) FFPE blocks were selected, and two 10 µm (H&A) and one 5 µm (FC) sections were sliced consecutively and mounted on regular (uncharged) glass slides by the Emory Neuropathology Core to enable easy removal of the tissue from the slide. Excess paraffin surrounding the tissue was removed by cutting around the tissue on the slide with a blade to improve RNA yield and quality. Following the removal of excess paraffin, the tissue on the slides was scraped into a tube. Total RNA from these two FFPE brain regions was isolated by the Emory Integrated Genomics Core using the TruXTRAC FFPE Total NA Plus Kit (Covaris LLC, 520255, Woburn, MA, USA), and automated on a KingFisher Flex instrument using the manufacturer’s protocol. RNA concentration and purity were measured using a Nanodrop One Spectrophotometer (Thermo Fisher Scientific, Waltham, MA, USA) and Bioanalyzer 2100 (Agilent Technologies, Santa Clara, CA, USA). RNA integrity (RIN) was determined by the Bioanalyzer 2100 (Agilent Technologies, Santa Clara, CA, USA). All RNA samples passed quality control and were processed for library preparation.

#### 4.3.2. Library Preparation and mRNA Sequencing

Using 15 cycles of PCR amplification, cDNA libraries were generated from 50ng of RNA using the SMART-Seq Stranded kit (Takara Bio Inc, 634444, Kusatsu, Shiga, Japan). Final libraries were quantitated by Qubit fluorometric quantitation (Thermo Fisher Scientific, Waltham, MA, USA) and size distribution determined by the Bioanalyzer 2100 (Agilent Technologies, Santa Clara, CA, USA). Successful libraries were pooled at equimolar ratios and then sequenced on the NovaSeq 6000 (Illumina, San Diego, CA, USA) by the Emory Integrated Genomics Core with PE75 configuration.

Samples generated between 4 and 382 million reads. Sequences were quality-checked by the Emory Integrated Computational Core using FastQC v.0.11.8, Fastq-Screen, and MultiQC v.12 [214] for completeness, depth, and read quality. Raw reads were trimmed using Trimmomatic v0.36 [215] and cleaned using cutadapt v3.4 [216]. The cleaned reads were aligned to the Sus scrofa (ss11.1) reference genome using STAR aligner v2.5.2 [217]. Alignments were sorted, filtered, and indexed to per-sample mRNA raw counts .bam files using samtools v1.3 [218]. Gene quantification or raw count generation from alignment .bam files was performed using HTSeq-count v0.13.5 [219].

#### 4.3.3. Filtering, Normalization, Outlier Removal, Differential Expression Analysis, and Visualization

RNA-seq counts were analyzed in R (v4.2.2). All swine gene names under the ss11.1 genome were retrieved via R annotation package org.Ss.eg.db, and the non-coding genes were removed. Samples with less than 10 million reads aligned were removed, and genes with less than 50 counts in more than 20 samples were filtered out to remove statistical background noise. Following filtering, 13,426 genes remained for normalization. Differences in quality measures, sequencing depth, and library size between injured 1-day-post-injury and Sham groups were present in our data. To adjust for these differences, read counts were normalized by the ratio of median method, then variance-stabilized using the DESeq2 package in R [220]. Outliers were detected and removed using the mahalanobis distance and multivariate outlier detection package in R [221]. Principle component analysis and expression of housekeeping genes, *RTF2*, *PPIB*, *YIPF3*, and *PSMB4* (https://housekeeping.unicamp.br), were evaluated to ensure data were adjusted and normalized efficiently. Following outlier removal and normalization, 68 samples (N = 40 subjects for one or both regions) remained for data analysis (Supplementary Table S1). The final group subject tallies for each region were as follows: (i) Sham (n = 8), (ii) injured 1-day survival (n = 9), (iii) injured 1-week survival (n = 7), and (iv) injured 1-day survival CsA-treated (n = 10). The raw and processed RNA-seq data for the 68 samples are available on the NCBI Gene Expression Omnibus (GEO) under accession number GSE274756.

DESeq2 differential expression analysis was performed using the DESeq2 package in R to identify genes that were significantly upregulated or downregulated between groups within each region. Wald’s test and Benjamini–Hochberg false discovery rate (FDR) procedures were implemented to perform hypothesis testing and adjustment for multiple comparisons. The cutoff for differentially expressed genes (DEGs) was performed at unadjusted *p* ≤ 0.05, log2FC ≥ 1 (upregulated) and log2FC ≤ -1 (downregulated) in all analyses. The top 5 DEGs were selected based on FDR ≤ 0.05 and the largest log2FC. Following DESeq2 analysis, volcano plots were generated using the EnhancedVolcano package in R. Heatmaps were generated using the heatmap3 and ComplexHeatmap packages in R. Pie charts, box plots, dot plots, bar charts, and Venn diagrams were generated using the ggplot2 and ggpubr packages in R. All plots were modified and/or created using Inkscape (v1.3.2) and BioRender.

### 4.4. Gene Ontology Analysis

Gene ontology (GO) overrepresentation analysis was employed to identify biological functions and relationships between DEGs [222]. Enrichment (statistical overrepresentation) analysis to measure which classes (GO biological process/GO terms) of genes were overrepresented was carried out in PANTHER (Protein Analysis Through Evolutionary Relationships, v18.0) [223]. The appropriate background was generated by using the genes that remained post-filtering. Fisher’s exact test, with the Benjamini–Hochberg false discovery rate (FDR) correction, identified statistically significant overrepresented terms (FDR-adjusted *p* ≤ 0.05). Using the ggplot2 package in R, bar plots were utilized to visualize overrepresented GO terms. For similar overrepresented GO terms within each comparison, the term with the highest fold enrichment was selected and visualized in bar plots.

### 4.5. Cell Type-Specific Gene Analysis

Our prior cell-type analyses of cell type-specific genes were obtained from Galea et al., 2022 [44], where they were identified by calculating the univariate cell-type enrichment τ score for every gene from data obtained from a multi-transcriptome study. Genes with τ ≥ 0.8 within a cell cluster were identified to be specific to that cell (i.e., cell type-specific), while genes τ ≥ 0.6 within a cell cluster were identified as being cell type-enriched [44]. All cell type-specific genes (τ ≥ 0.8) for neurons, astrocytes, microglia, oligodendrocytes, and endothelial cells were extracted and utilized for this analysis.

### 4.6. Weighted Gene Co-Expression Network Analysis (WGCNA)

Weighted gene co-expression network analysis (WGCNA) was performed on the 13,426 genes within each comparison using the WGCNA package in R [224,225]. This was carried out to study gene interactions by identifying clusters (modules) of highly correlated genes. Firstly, we constructed our network in a single block using the blockwiseModules() function. Due to high variability in our data, a signed network soft thresholding power β of 12 was selected, as recommended by the WGCNA FAQ, based on a sample size of more than 40. This soft thresholding power β was selected to improve the co-expression similarity and calculate the adjacency. The minimum number of genes in the module was 10, the module detection sensitivity was 4 (deepSplit = 4), the cut height for merging of modules was 0.15 (mergeCutHeight = 0.15), the reassignment threshold was 0.05, and a “bicor” correlation type and a “mean” TOM denominator were chosen. These parameters were utilized to identify our module eigengenes (MEs) and their expression scores. To compare ME expression scores between Sham, injured 1-day survival, injured 1-week survival, and injured CsA-treated groups, a Wilcox test and Bonferroni adjustment were applied (*p* ≤ 0.05).

### 4.7. Axonal Pathology (Percent Axonal Injury)

The extent of axonal injury was measured via APP staining, as outlined in Naim et al., 2011 [83] and Weeks et al., 2014 [84]. Briefly, 6 µm thick coronal sections were obtained at 3mm intervals throughout the entire brain and stained for axonal injury (β-amyloid precursor protein, β-APP). Regions of microscopic axonal injury were outlined in each section by a neuropathologist (C. Smith). Finally, the percent of axonal injury was calculated by dividing the total outlined area of injured white matter in the sections by the total brain area. To compare the percentage of axonal injury between Sham, injured 1-day survival, and injured 1-week survival groups, a Wilcox test and Bonferroni adjustment were applied (*p* ≤ 0.05).

## 5. Conclusions

Utilizing an immature swine model of diffuse mTBI, we identified an increased abundance of DEGs from 1 day post-injury to 1 week post-injury in the FC and H&A. At 1 day post-injury, we observed neuronal distress, which was characterized by a Transient and Early downregulation of cell survival genes in both regions, and upregulation of Persistent neuron degenerative and protective DEGs in both regions. These changes were coupled with a Delayed and Late emergence of decreased microglia-specific genes and increased neuron- and astrocyte-specific genes associated with neurorecovery at 1 week post-injury in both regions, and an Intensified upregulation of cell survival genes in the H&A above 1-day increases. Finally, we report that after only 1 day, CsA treatment accelerated neurorecovery-associated genes rather than inhibiting the early neuronal distress genes we identified that were altered at 1 day post-injury without treatment. Overall, our data reveal the effects of anatomic region and elapsed time on gene expression post-mTBI and motivate future mechanistic and interventional studies for CsA in mTBI.

## Figures and Tables

**Table 1:** Ranges of top 5 upregulated and downregulated DEGs in both regions.

	Time Post-Injury	FC Log2FC	H&A Log2FC
Downregulated	1 day	−1.68 to −1.58	−1.80 to −1.70
	1 week	−2.28 to −2.00	−2.75 to −2.46
Upregulated	1 day	1.53 to 1.89	1.85 to 2.10
	1 week	1.69 to 2.46	2.00 to 2.09

Data are presented as ranges for all variables. Log2FC, log2 fold change. FC, frontal cortex. H&A, hippocampus + amygdala.

**Table 2:** Body weight, rotational velocity, and rotational acceleration of piglets.

Group	Body Weight (kg)	Velocity (rad/s)	Acceleration (krad/s^2^)
Anesthesia-only Sham	9.3 ± 1.5	-	-
Naïve	8.6 ± 1.4	-	-
Injured 1-day survival	8.3 ± 0.7	124.3 ± 1.7	52.0 ± 8.1
Injured 1-week survival	9.4 ± 1.5	124.7 ± 3.3	40.8 ± 6.9
Injured 1-day survival CsA-treated	8.2 ± 0.8	124.8 ± 2.5	59.4 ± 9.7

Data are presented as mean ± standard deviation (SD) for all variables.

## Data Availability

All raw and processed RNA-seq datasets generated and analyzed in this study are available in the NCBI Gene Expression Omnibus (GEO) repository under accession number GSE274756. Source values for all figures are provided with this paper in Supplementary tables.

## Supplementary Materials

The supporting information can be downloaded at https://www.mdpi.com/article/10.3390/ijms26083531/s1.

## Abbreviations

The following abbreviations are used in this manuscript:

mTBI	mild traumatic brain injury
FFPE	formalin-fixed paraffin-embedded
FC	frontal cortex
H&A	hippocampus + amygdala
RNR	rapid non-impact head rotation
CsA	cyclosporine A
DEGs	differentially expressed genes
Log2FC	log2 fold change
FDR	false discovery rate
RNA-seq	RNA sequencing
GO	gene ontology
WGCNA	weighted gene co-expression network analysis
ME	module eigengene
