# Supplementary material for: Cyclosporine A Accelerates Neurorecovery Transcriptional Trajectory in a Swine Model of Diffuse Traumatic Brain Injury
Source: Int J Mol Sci. 2025 Apr 9;26(8):3531. doi: 10.3390/ijms26083531 (PMC12026708; doi:10.3390/ijms26083531)
Supplement: Supplementary file 1 [file ijms-26-03531-s001.zip › Supplementary Materials/Supplementary Information.docx]

**Supplementary Information**

*Article*

**Cyclosporine A Accelerates Neurorecovery Transcriptional Trajectory in a Swine Model of Diffuse Traumatic Brain Injury**

Oluwagbemisola Aderibigbe^1^, Levi B. Wood^1,2*^, Susan S. Margulies^1*^

**Affiliations:**

^1^Wallace H. Coulter Department of Biomedical Engineering, Georgia Institute of Technology and Emory University, Atlanta, Georgia, USA

^2^George W. Woodruff School of Mechanical Engineering and Parker H. Petit Institute for Bioengineering and Bioscience, Georgia Institute of Technology, Atlanta, Georgia, USA

* Authors for correspondence:

Levi B. Wood, Ph.D. (Email: [levi.wood@me.gatech.edu](mailto:levi.wood@me.gatech.edu))

Susan S. Margulies, Ph.D. (Email: [susan.margulies@gatech.edu](mailto:susan.margulies@gatech.edu))

**Supplementary Figures**


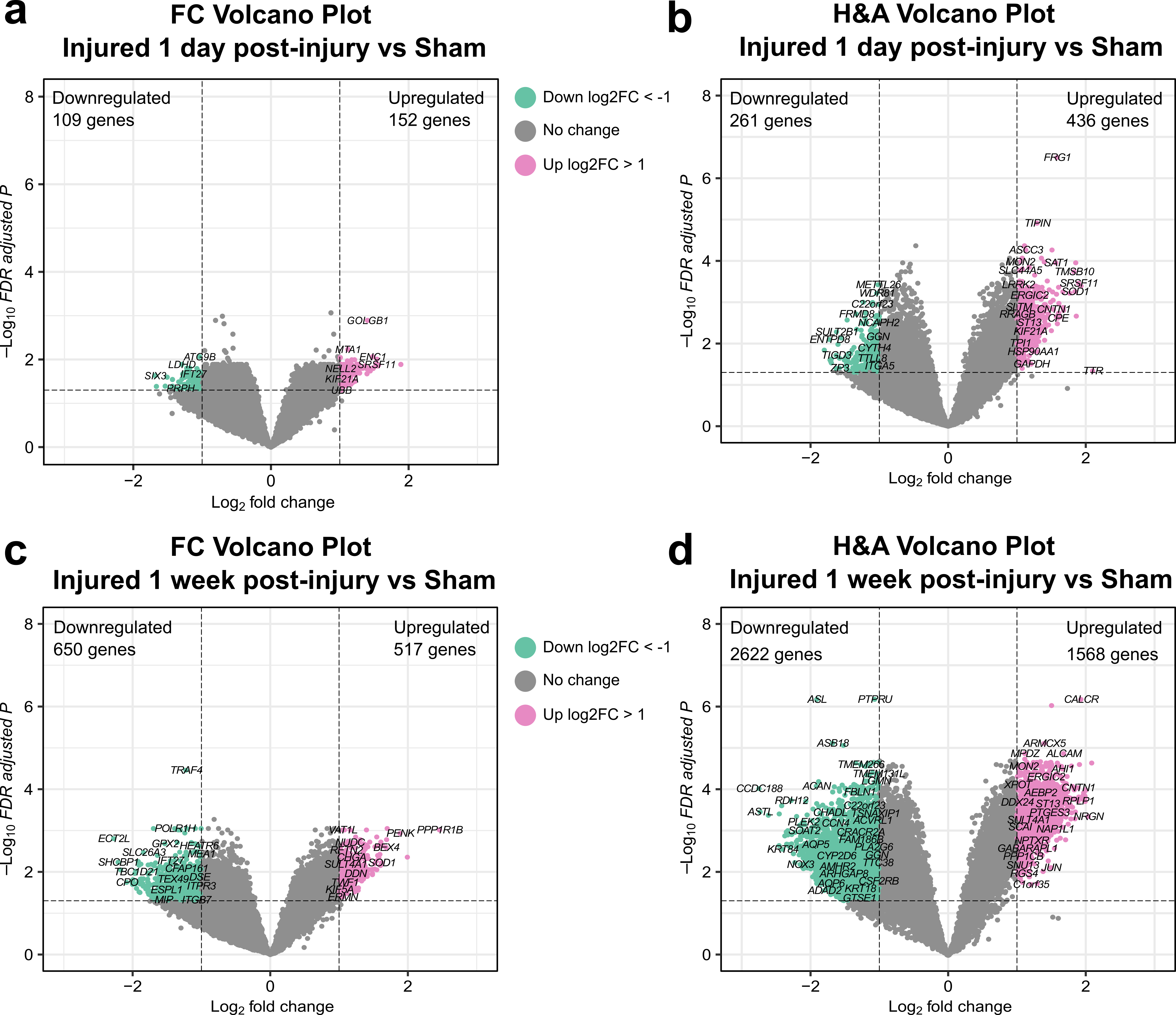


**Supplementary Figure S1** FDR adjusted differentially expressed genes (DEGs). Volcano plot of differentially expressed genes (DEGs) between Sham and injured 1 day post-injury group in the **(a)** FC and **(b)** H&A, consecutively. DEGs have FDR ≤ 0.05 (above dashed horizontal line) and corresponding log2 fold change |log2FC| ≥ 1. DEGs between Sham and injured 1 week post- injury group in the **(c)** FC and **(d)** H&A, consecutively. DEGs have FDR ≤ 0.05 (above dashed horizontal line) and corresponding log2 fold change |log2FC| ≥ 1. DEGs, differentially expressed genes. Log2FC, log2 fold change. FDR, false discovery rate. FC, frontal cortex. H&A, hippocampus + amygdala


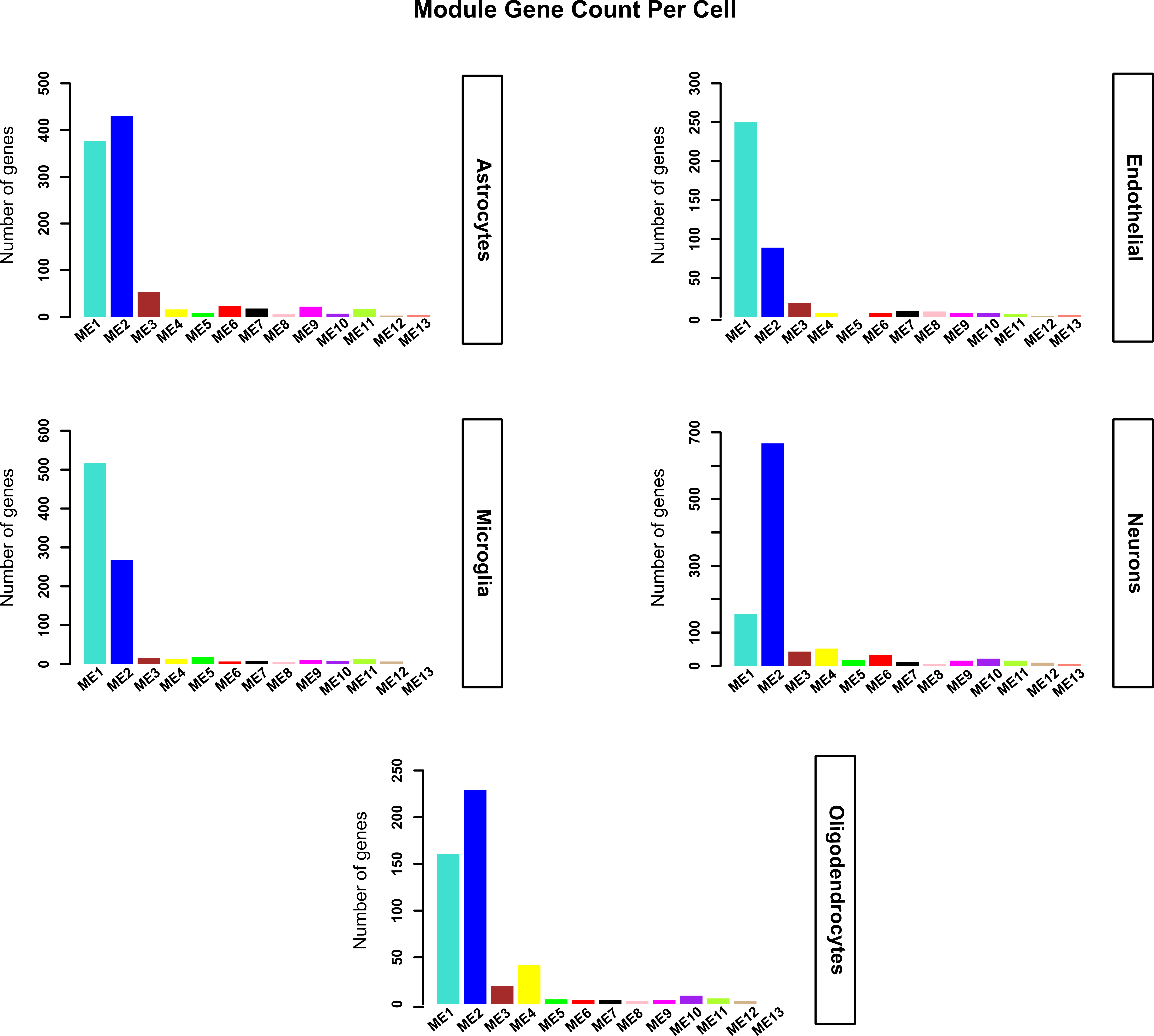


**Supplementary Figure S2** Cell type specific analysis for WGCNA modules. The number of genes within each module that are astrocyte, endothelial, microglia, neurons, and oligodendrocyte specific.


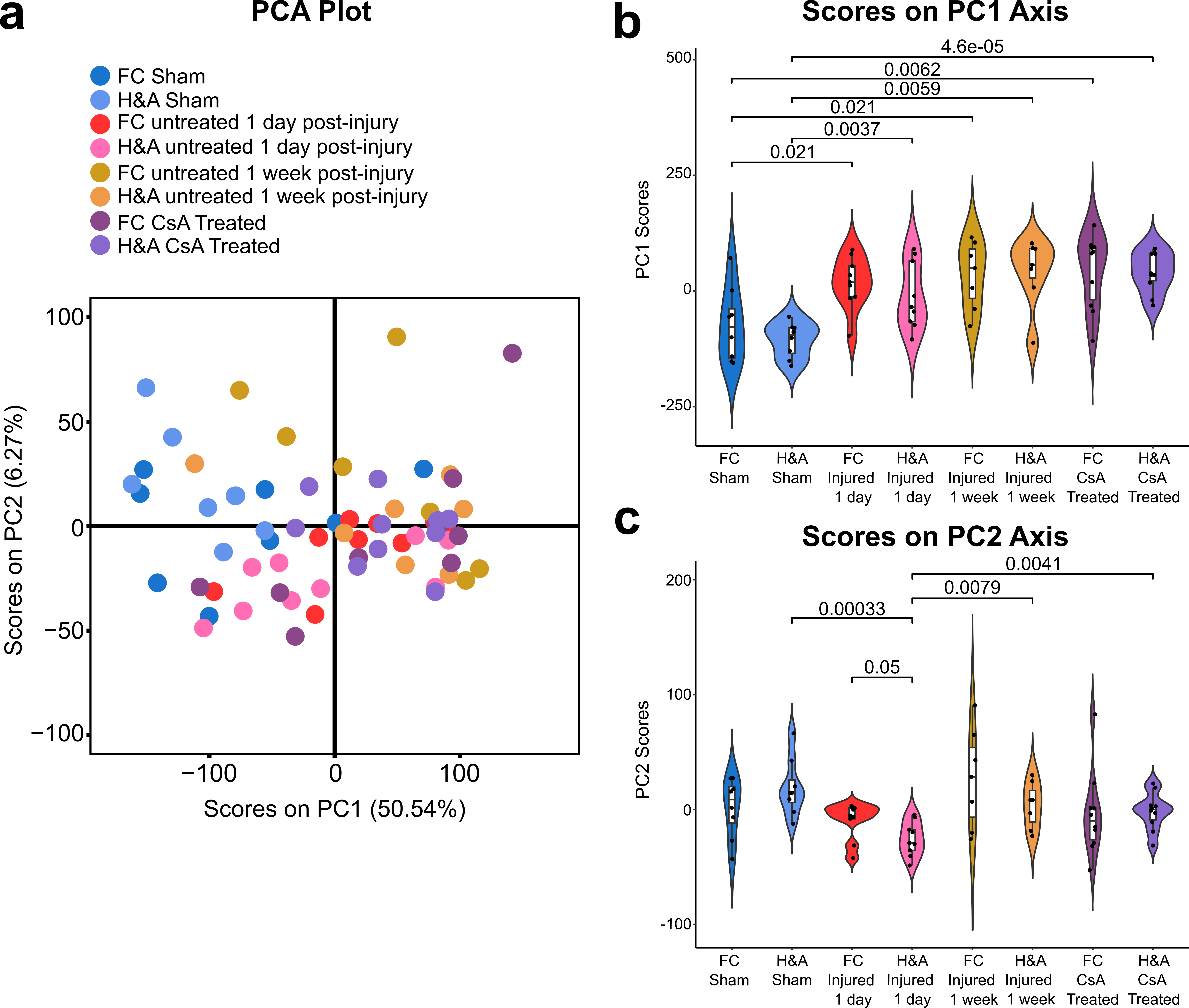


**Supplementary Figure S3** Principal component analysis of all groups. **(a)** Principal component analysis (PCA) of genes across Sham, injured 1 day post-injury, injured 1 week post-injury, and Cyclosporine treated groups in the frontal cortex and hippocampus + amygdala. **(b)** Violin plot of PC1 and **(c)** PC2 scores showing distribution of data within all four groups in the frontal cortex (FC) and hippocampus + amygdala (H&A) (Wilcox test, Bonferroni adjusted p ≤ 0.05). PC1, first principal component. PC2, second principal component. FC, frontal cortex. H&A, hippocampus + amygdala. CsA, Cyclosporine A.


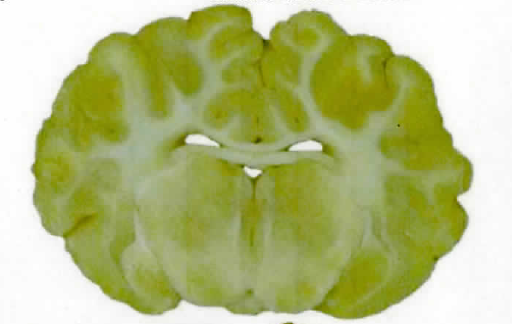

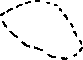


A

H

**Supplementary Figure S4** Hippocampus + Amygdala regions. The area in the dotted lines represent the typical section removed for the hippocampus + amygdala (H&A). A, amygdala. H, hippocampus.

**Supplementary Tables**

**Supplementary Tables S1 – 7 and their descriptions.**

| **Supplementary Table Number** | **Description** |
| --- | --- |
| Supplementary Table S1 | Animal subjects utilized in study for each step of data workflow and percent axonal injury data |
| Supplementary Table S2 | Cell type specific downregulated and upregulated DEGs in the frontal cortex and hippocampus + amygdala for each temporal pattern |
| Supplementary Table S3 | Transient, Early, Intensified, and Persistent DEGs in the frontal cortex and hippocampus + amygdala |
| Supplementary Table S4 | Delayed and Late DEGs in the frontal cortex and hippocampus + amygdala |
| Supplementary Table S5 | Common downregulated and upregulated DEGs across time and region. |
| Supplementary Table S6 | Dampened and accelerated downregulated and upregulated DEGs |
| Supplementary Table S7 | 13 functional modules from the WGCNA analysis |
| Supplementary Table S8 | DEGs from all comparisons in the frontal cortex and hippocampus + amygdala |
