# Supplementary material for: Cyclosporine A Accelerates Neurorecovery Transcriptional Trajectory in a Swine Model of Diffuse Traumatic Brain Injury
Source: Int J Mol Sci. 2025 Apr 9;26(8):3531. doi: 10.3390/ijms26083531 (PMC12026708; doi:10.3390/ijms26083531)
Supplement: Supplementary file 1 [file ijms-26-03531-s001.zip › Supplementary Materials/Supplementary Information.pdf]

Supplementary Information

Article

Cyclosporine A Accelerates Neurorecovery Transcriptional Trajectory in a Swine Model of Diffuse Traumatic Brain Injury

Oluwagbemisola Aderibigbe[1], Levi B. Wood[1,2*], Susan S. Margulies[1*]

Affiliations:

[1]Wallace H. Coulter Department of Biomedical Engineering, Georgia Institute of Technology and Emory University, Atlanta, Georgia, USA

[2]George W. Woodruff School of Mechanical Engineering and Parker H. Petit Institute for Bioengineering and Bioscience, Georgia Institute of Technology, Atlanta, Georgia, USA

* Authors for correspondence:
Levi B. Wood, Ph.D. (Email: levi.wood@me.gatech.edu)
Susan S. Margulies, Ph.D. (Email: susan.margulies@gatech.edu)

Supplementary Figures

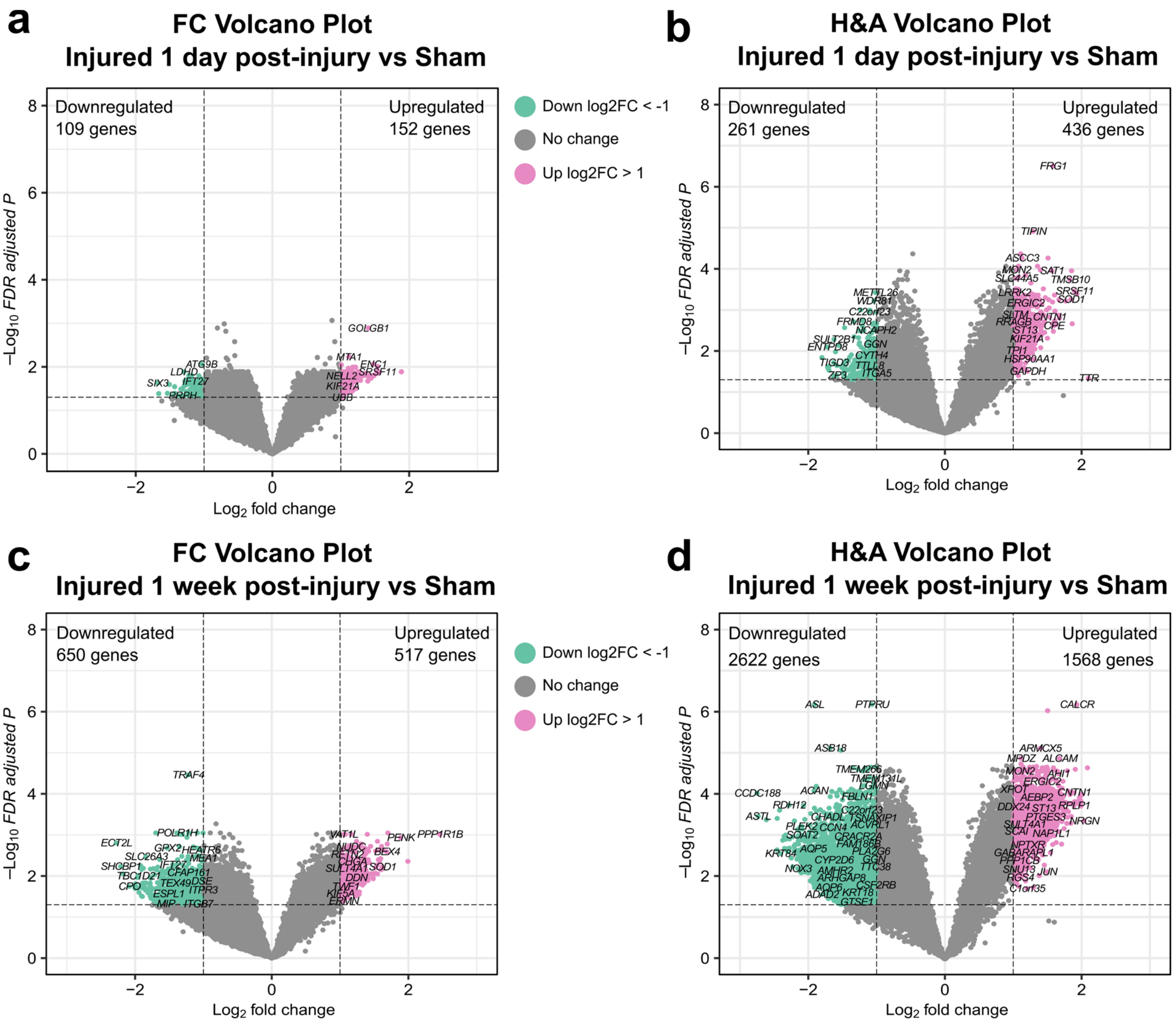

Supplementary Figure S1 FDR adjusted differentially expressed genes (DEGs). Volcano plot of differentially expressed genes (DEGs) between Sham and injured 1 day post-injury group in the **(a)** FC and **(b)** H&A, consecutively. DEGs have FDR ≤ 0.05 (above dashed horizontal line) and corresponding log2 fold change |log2FC| ≥ 1. DEGs between Sham and injured 1 week post- injury group in the **(c)** FC and **(d)** H&A, consecutively. DEGs have FDR ≤ 0.05 (above dashed horizontal line) and corresponding log2 fold change |log2FC| ≥ 1. DEGs, differentially expressed genes. Log2FC, log2 fold change. FDR, false discovery rate. FC, frontal cortex. H&A, hippocampus + amygdala

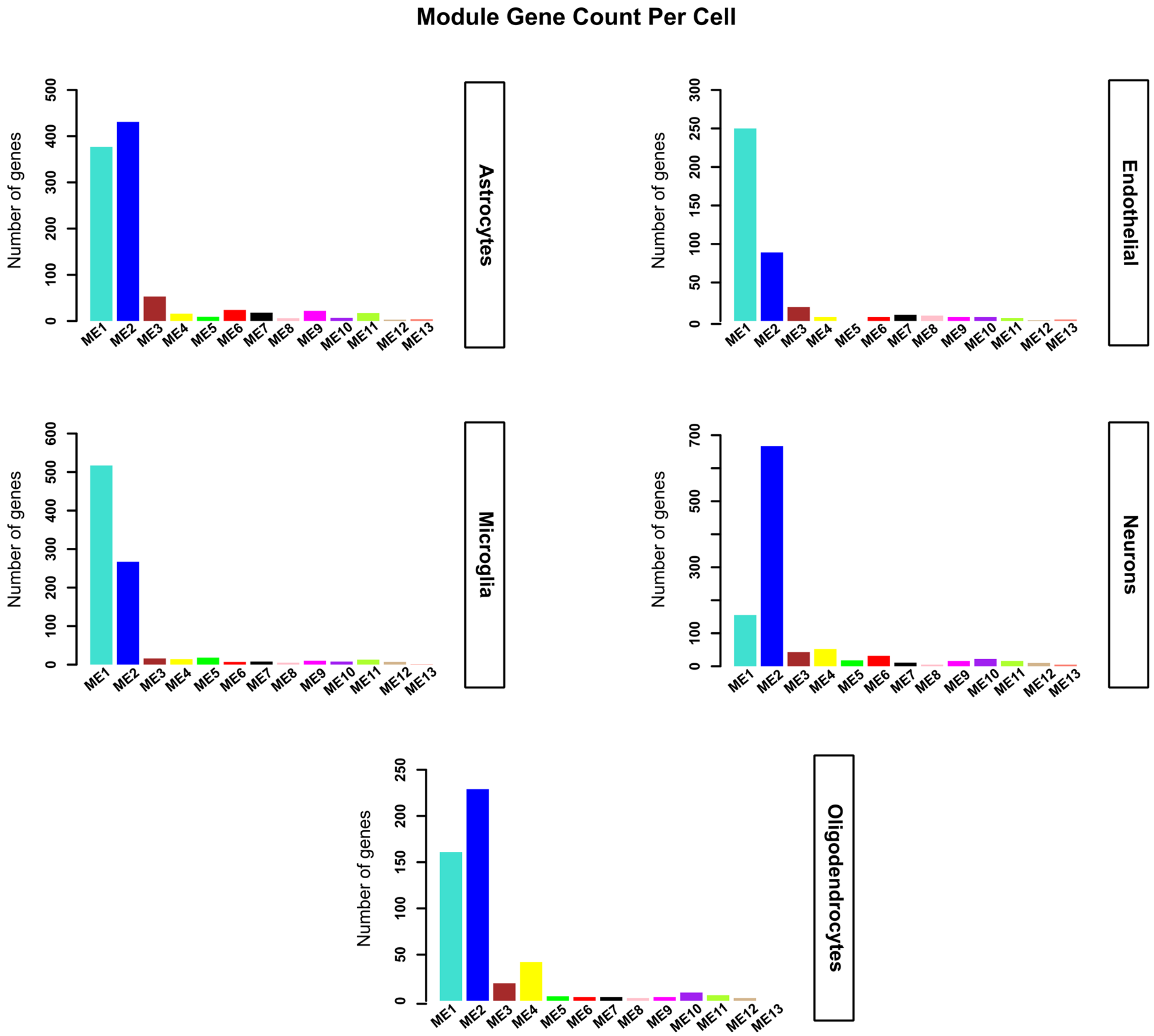

Supplementary Figure S2 Cell type specific analysis for WGCNA modules. The number of genes within each module that are astrocyte, endothelial, microglia, neurons, and oligodendrocyte specific.

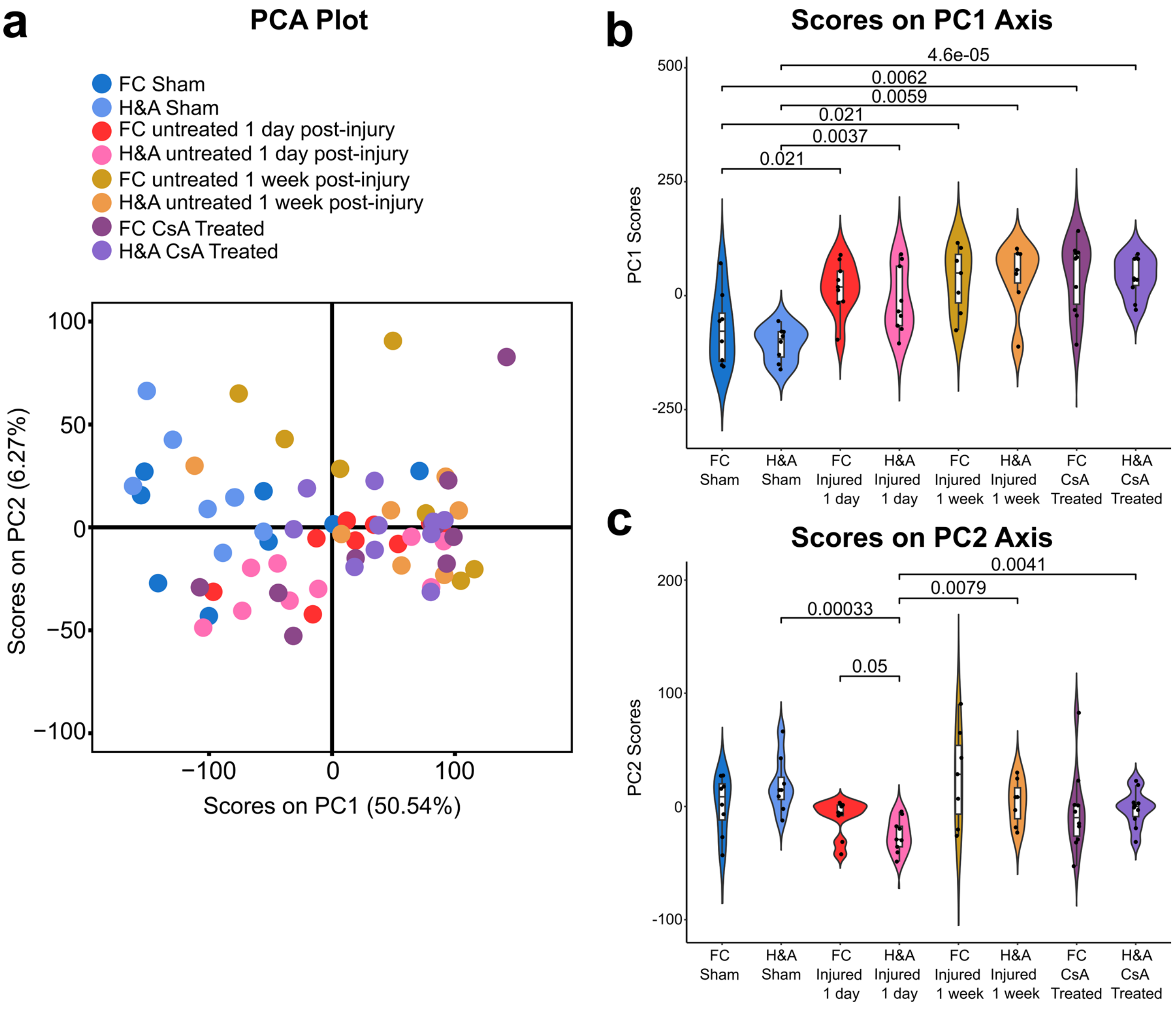

Supplementary Figure S3 Principal component analysis of all groups. **(a)** Principal component analysis (PCA) of genes across Sham, injured 1 day post-injury, injured 1 week post-injury, and Cyclosporine treated groups in the frontal cortex and hippocampus + amygdala. **(b)** Violin plot of PC1 and **(c)** PC2 scores showing distribution of data within all four groups in the frontal cortex (FC) and hippocampus + amygdala (H&A) (Wilcox test, Bonferroni adjusted $p \leq 0.05$). PC1, first principal component. PC2, second principal component. FC, frontal cortex. H&A, hippocampus + amygdala. CsA, Cyclosporine A.

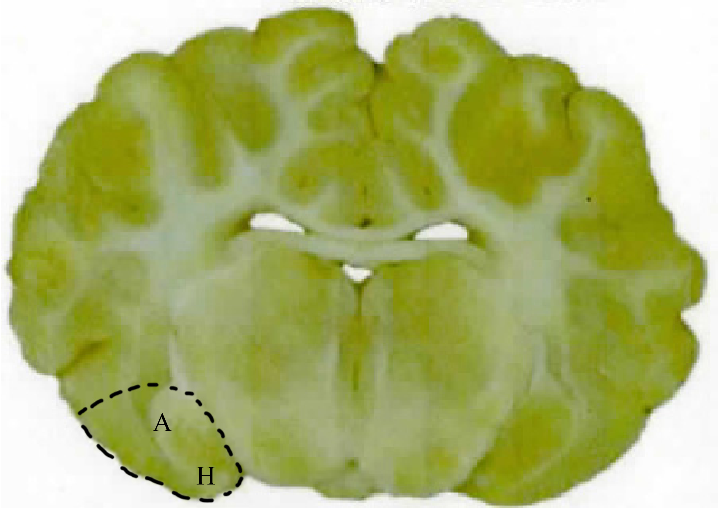

Supplementary Figure S4 Hippocampus + Amygdala regions. The area in the dotted lines represent the typical section removed for the hippocampus + amygdala (H&A). A, amygdala. H, hippocampus.

Supplementary Tables

Supplementary Tables S1 – 7 and their descriptions.

**Supplementary Table Number**	**Description**
Supplementary Table S1	Animal subjects utilized in study for each step of data workflow and percent axonal injury data
Supplementary Table S2	Cell type specific downregulated and upregulated DEGs in the frontal cortex and hippocampus + amygdala for each temporal pattern
Supplementary Table S3	Transient, Early, Intensified, and Persistent DEGs in the frontal cortex and hippocampus + amygdala
Supplementary Table S4	Delayed and Late DEGs in the frontal cortex and hippocampus + amygdala
Supplementary Table S5	Common downregulated and upregulated DEGs across time and region.
Supplementary Table S6	Dampened and accelerated downregulated and upregulated DEGs
Supplementary Table S7	13 functional modules from the WGCNA analysis
Supplementary Table S8	DEGs from all comparisons in the frontal cortex and hippocampus + amygdala
